# ZIF-67 and Mn & Zn co-doped ZIF-67 as multifunctional therapeutic platforms: Antibacterial, antioxidant, and anticancer assessment in lung cancer cells *in vitro*

**DOI:** 10.1016/j.bbiosy.2026.100148

**Published:** 2026-09-19

**Authors:** Preema Cealla Pais, Ronald Aquin Nazareth, Ashwini Prabhu, Sudhakar Y N, Arnab Parui

**Affiliations:** a Department of Chemistry, St. Aloysius (Deemed to be University), Mangaluru 575003 Karnataka, India; b Division of Cancer Research and Therapeutics (CaRT), Yenepoya Research Centre, Yenepoya (Deemed to be University), Mangalore 575 018, India; c Central Research Laboratory, Srinivas Institute of Medical Sciences and Research Centre, Srinivas University, Mangalore 574146, India; d Manipal Institute of Technology, Manipal Academy of Higher Education, Manipal, India

**Keywords:** Doped metal organic frameworks, Antibacterial, Antioxidant, Anticancer, Apoptosis

## Abstract

The present study is an attempt to delineate the biological efficacy of zinc and manganese doping on ZIF-67 MOF. Pure (ZIF-67) and zinc/manganese-doped samples were synthesised using a one-pot method. Various analytical techniques such as fourier transform infrared spectroscopy (FTIR), X-ray diffraction (XRD), field emission scanning electron microscopy (FESEM), transmission electron microscopy (TEM), dynamic light scattering (DLS) together with elemental mapping analyses through energy dispersive spectroscopy (EDS), support that the MOF formed is a sodalite (SOD) cage type structure. The study of surface properties through Brunauer-Emmett-Teller (BET) analysis unfolded the formation of microporous pristine MOF and mesoporous bimetallic MOF compounds with a surface area of 1227.4 and 4.3 m^2^g^−1^ respectively. Additionally, thermal gravimetric analysis (TGA) depicted that the codoped sample decomposed about 20% at 700°C when compared to its precursor which only retained 20% mass at the same temperature. The X-ray Photoelectron spectroscopy (XPS) results showed effective dopant incorporation and strong interfacial interactions between the dopant ions and the MOF framework. ICP-OES studies demonstrated the incorporation of zinc and manganese into the ZIF framework Furthermore, all the doped samples displayed exceptional antimicrobial activity against *Staphylococcus aureus* and *Escherichia coli* and also exhibited enhanced antioxidant activity in comparison to their precursor. Sample Z showed the strongest antibacterial activity against *E. coli* (MIC 31.25 µg/mL; MBC 250 µg/mL), while Zn/Mn modification enhanced the activity against *S. aureus*, lowering the MIC from 500 to 250 µg/mL for ZM and ZMZ. The *in-vitro* anticancer study performed on human lung adenocarcinoma cells (A549) revealed the enhancement in the antiproliferative potential of bimetallic Zn/Mn doped ZIF-67 MOFs in comparison to pure ZIF-67 nanostructures. In addition, the orange-red fluorescence observed in A549 nuclei during AO-EB staining indicated late apoptosis induced by the nanocomposites, and complete nuclear condensation coupled with fragmentation, as visualized by Hoechst staining. Thus, the studies have demonstrated the superior cytotoxic and antimicrobial potential of monometal and bimetal doped ZIF-67 samples.

## Highlights

• Bimetallic Zn/Mn doping tailors ZIF structures to enhance multifunctional bioactivity.
• Doped ZIF exhibit superior antimicrobial and antioxidant efficiency overall.
• Zn/Mn codoping improves thermal stability and pore features enabling biomedical impact.
• Modified ZIF nanostructures induce cancer cell apoptosis showing strong therapeutic promise.

## Graphical abstract

**Figure:**
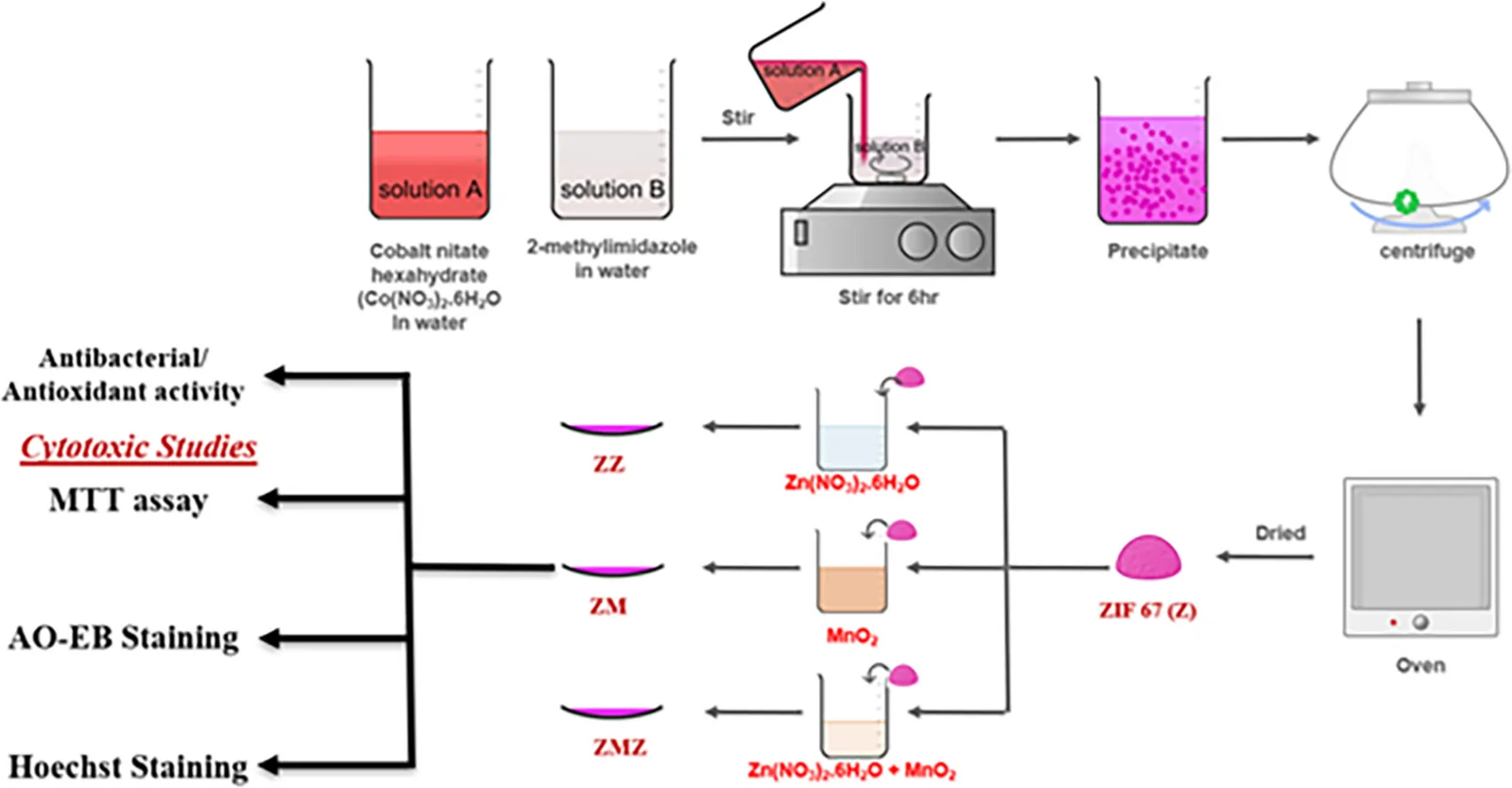

## 1 Introduction

Metal-organic frameworks (MOFs) are an important new class of coordinated micro or nanopore networks with exceptional characteristics. This is because of their ability to control internal porosity, surface chemistry, and particle size, resulting in the creation of more intricate materials [1,2] .These crystalline frameworks combine adjustable pore structures with very high surface areas and substantial pore volumes, offering a level of functional versatility that conventional materials rarely achieve [3]. As their synthesis can be systematically customized, it is possible to fine-tune the topology, pore architecture, and chemical environment of MOFs, positioning them as an exceptionally adaptable class of nanomaterials for diverse applications [4].

Zeolitic imidazolate frameworks (ZIFs) constitute an important subclass of metal–organic frameworks in which tetrahedrally coordinated transition-metal centres, commonly Zn²⁺ or Co²⁺, are interconnected by imidazolate-based linkers. The imidazolate bridges generate an M–Im–M connectivity angle of approximately 145°, closely approximating the Si–O–Si angle characteristic of many zeolitic frameworks. This geometric analogy enables ZIFs to adopt zeolite-like topologies while retaining the compositional versatility, tunable porosity, and functional diversity [[5], [6], [7]]. Unlike conventional zeolites, ZIFs can be synthesized under relatively mild conditions, including at room temperature, further enhancing their practical utility [8].Their modular nature allows for functionalization through ligand variation, metal substitution, or post-synthetic modification, enabling the design of ZIFs for specific applications. For instance, ZIF-8 (Zn-based MOF) has been employed for gas separation and drug delivery due to its biocompatibility, while ZIF-9 and ZIF-68 demonstrate potential in hydrocarbon separations and catalysis. A significant feature of ZIFs is their ability to integrate into composite materials with polymers, carbon nanostructures, or metal nanoparticles, thereby enhancing conductivity and stability [9].

Among various ZIFs, ZIF-67 is characterized by an enriched surface area of (S_BET_ ​ > 1700 m² g⁻¹), which provides a large density of accessible active sites. Moreover, its intrinsic microporous structure, with an average pore diameter of approximately 0.34 nm, facilitates strong interactions with guest molecules, thereby promoting efficient adsorption and reaction processes. Its combination of high porosity, chemical and thermal stability (decomposition temperatures reported near ≈350 °C in some preparations), and facile functionalization makes ZIF-67 attractive for adsorption, catalysis, sensing, membrane applications, and biomedical research [10,11]. In accordance with biomedical research, multiple studies report potent antibacterial and antibiofilm activity of ZIF-67 nanoparticles against clinically relevant pathogens [12,13].

Among the diverse MOF materials, cobalt-based metal–organic frameworks (Co-MOFs) have attracted considerable interest for biological and biomedical applications, and proven potential for structural and surface functionalization. These characteristics enable Co-MOFs to be engineered for applications including antimicrobial therapy, drug delivery, biosensing, and other therapeutic strategies [[14], [15], [16]]. For instance, ZIF-67 nanosheets exhibit intrinsic peroxidase-mimetic activity, successfully catalyzing the oxidation of 3,3′,5,5′-tetramethyl-benzidine (TMB) in the absence of H₂O₂, highlighting its potential as a colorimetric biosensing probe. In a separate study, ZIF-67 nanoparticles demonstrated potent antibacterial and antibiofilm activity against major pathogens such as *Acinetobacter baumannii, Pseudomonas aeruginosa*, and *Staphylococcus aureus*, whilst maintaining low cytotoxicity in mammalian cell models, underscoring its promise for antimicrobial surface coatings or therapeutics [17,18]. In addition, ZIF-67 has been employed as a high‐capacity carrier for enzyme immobilization: a study showed that phospholipase A1 purely immobilised on ZIF-67 reached a loading capacity of ∼619.8 mg/g and retained ∼130% of the free enzyme activity, enabling efficient conversion of phospholipids into docosahexaenoic acid (DHA)-enriched lipids for nutritional and bioactive applications. Collectively, these examples illustrate ZIF-67 as a multifunctional unit serving as a catalytic nanozyme, antimicrobial agent and biocatalyst support thus making it highly attractive for diverse bio-related fields ranging from diagnostics and therapeutics to biomanufacturing [[19], [20], [21]].

Recent studies indicate that ZIF-67-based systems can also promote reactive oxygen species (ROS)-associated cancer-cell damage and exhibit enzyme-mimicking redox activity, supporting their potential in anticancer applications and antioxidant-related sensing. Furthermore, functionalization or incorporation of bioactive components into ZIF-67 can enhance its antioxidant and therapeutic performance. These findings highlight ZIF-67 as a tunable platform whose biological activity can be modulated through framework composition and functional modification [[22], [23], [24]]. Modifying ZIF-67 can significantly broaden its biological potential [25]. For example, Ag incorporation enhanced the antibacterial performance of ZIF-67, whereas polydatin-functionalized ZIF-67 showed improved bacterial inhibition through ROS generation and membrane damage [26,27]. Existing literature on TiO₂/ZIF-67 systems demonstrated that modification of ZIF-67 can significantly influence its antimicrobial performance. The study showed that the antibacterial activity against *Staphylococcus aureus* was strongly dependent on the structural characteristics and solubility of the modified ZIF-67 material, with the most effective composite exhibiting pronounced biocidal activity at 2.5 mg/mL. The authors suggested that metal-ion/ligand release together with redox-mediated processes may contribute to bacterial inactivation, highlighting the importance of framework modification in tuning the biological behavior of ZIF-67 [28]. In a study, resveratrol-functionalized ZIF-67 exhibited cytotoxicity toward colon cancer cells and reduced their migratory and colony-forming abilities [29]. In another approach, Mn-containing modification of ZIF-67 enabled glutathione depletion and combined chemodynamic and photothermal effects, resulting in enhanced tumor inhibition [30].

In this study, ZIF-67 and Zn/Mn-codoped metal-organic frameworks (MOFs) were synthesized through a straightforward ambient-temperature solvent mixing process. Following comprehensive characterization, these nanostructures were assessed for their therapeutic potential. The experimental data confirmed that the MOFs possess significant radical-scavenging, antimicrobial, and anticancer capabilities while maintaining good biocompatibility. Notably, the bimetallic Zn/Mn-ZIF-67 structures exhibited good structural integrity and biological efficacy compared to their monometallic ZIF-67 counterparts. Although Zn and Mn-doped ZIF-67 systems have previously been investigated for antibacterial and anticancer applications the novelty of the present study lies in the development of our specific engineered codoped ZIF-67-based formulation, its comprehensive physicochemical characterization, and the demonstration of both antibacterial activity and selective toxicity on the cancer cells, We have further strengthened the mechanistic aspect by including western blot analysis of Bax, Bcl-2, and caspase-3, providing experimental evidence for apoptosis, and by evaluating cytocompatibility in BEAS-2B normal lung epithelial cells, which demonstrates selective toxicity toward cancer cells (Fig. 1).

**Fig. 1:**
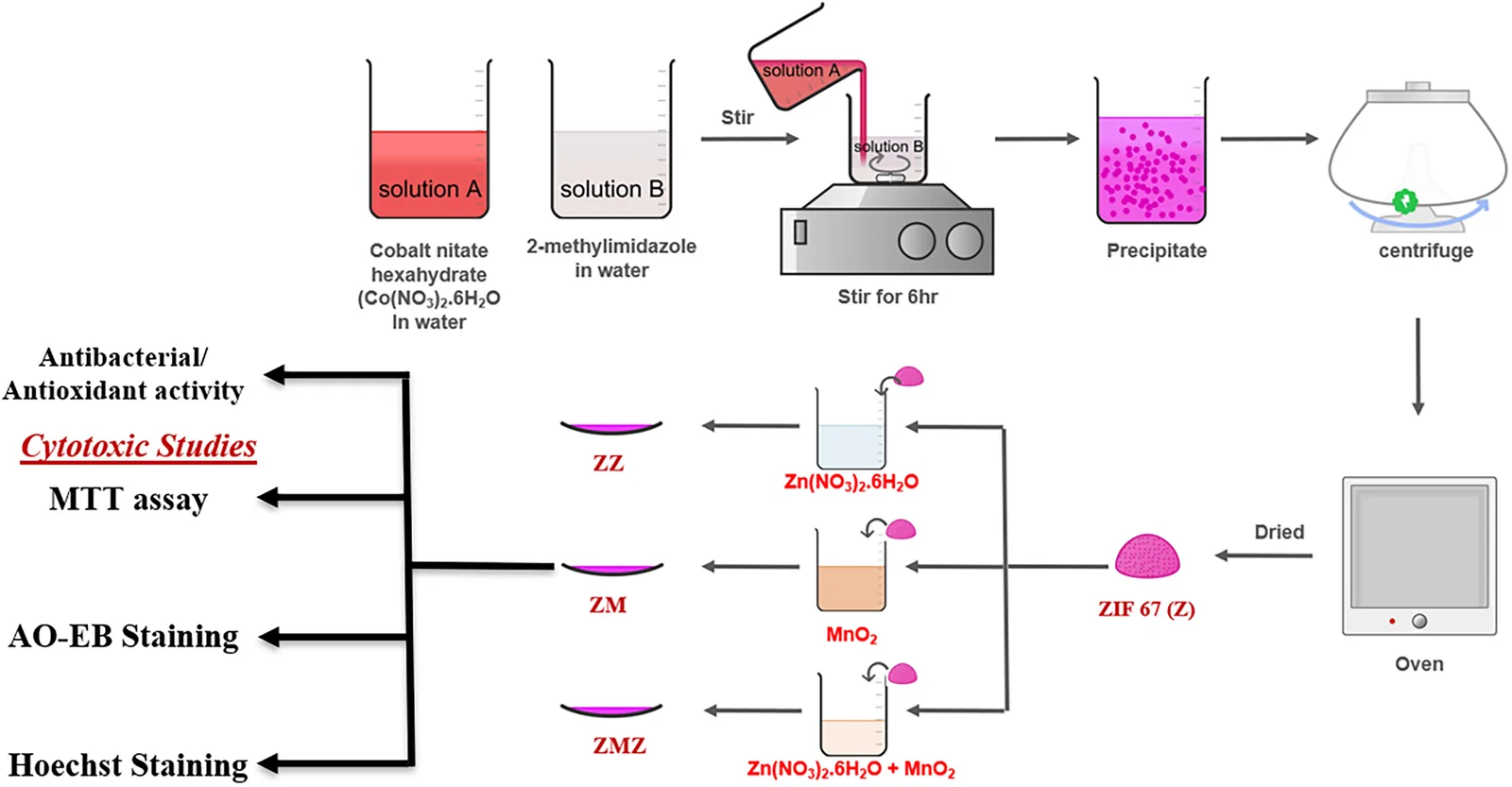
Schematic flowchart illustrating the synthesis and applications of ZIF-67 and its doped samples.

## 2 Materials and methods

### 2.1 Materials

Analytical-grade chemicals were used as received without further purification. Cobalt nitrate hexahydrate, (Co(NO_3_)_2_⋅6H_2_O ≥99.5%), ethanol (≥99.9%) were procured from Merck, India. Methyl imidazole (99%), Zinc nitrate hexahydrate (Zn(NO_3_)_2_⋅6H_2_O, 98%), Manganese dioxide (MnO_2,_ ≥97%), were procured from SRL, India. 2,2-Diphenyl-1-picrylhydrazyl (DPPH), and DMSO were obtained from Merck-India. Dulbecco’s Modified Eagle’s Medium (DMEM), foetal bovine serum (FBS), L-Glutamine, Antibiotic-antimycotic solution, and Trypsin-EDTA were procured from Gibco, Thermo Fisher Scientific. MTT, acridine orange, ethidium bromide, and Hoechst were procured from HiMedia, India.

### 2.2 Synthesis of pristine MOF and its doped samples

#### 2.2.1 Synthesis of ZIF-67 (Z)

ZIF-67 was synthesised using a slightly modified version of a previously published procedure [31]. 13.04% of cobalt nitrate hexahydrate was dissolved in 3 mL of double-distilled water (solution A), and 21.57% of 2-methylimidazole was dissolved in 20 mL of double-distilled water (solution B). Solution A was added to solution B, which was continuously mixed for 6 h at room temperature. The purple precipitate was recovered by centrifugation, washed thoroughly with distilled water several times, and dried overnight at 80 °C to produce the ZIF-67 powder.

#### 2.2.2 Synthesis of Zn doped ZIF-67 (ZZ)

4.63% of ZIF-67 and 0.46% of Zinc nitrate hexahydrate is mixed in a beaker. To this, 2.6 ml of ethanol is added. It is mixed for 1 h at room temperature and dried overnight.

#### 2.2.3 Synthesis of Mn doped ZIF-67 (ZM)

4.63% of ZIF-67 and 0.46% of manganese dioxide are mixed in a beaker. To this, 2.6 mL of ethanol is added, and the mixture is stirred for 1 h at room temperature. The residue obtained is dried overnight.

#### 2.2.4 Synthesis of Zn and Mn doped ZIF-67 (ZMZ)

To a mixture of 4.63% of ZIF-67, 0.46% of zinc nitrate hexahydrate was added and transferred into a 100 mL beaker. To this 0.46% of manganese dioxide is added and is dispersed in 6 mL of ethanol. The solution was magnetically stirred at room temperature for 1 h to ensure uniform mixing. After stirring, the magnetic bead was removed, and the resulting mixture was allowed to dry overnight at room temperature.

The samples and sample codes assigned are depicted as follows ZIF-67 is assigned the code Z, Zn doped ZIF-67 as ZZ, Mn doped ZIF-67 as ZM, Zn/Mn codoped ZIF- 67 as ZMZ.

### 2.3 Evaluation of the antibacterial activity

*In vitro* antimicrobial activity of the samples Z, ZZ, ZM, ZMZ were tested against *Staphylococcus aureus*, a Gram-positive bacterium, and *Escherichia coli*, a Gram-negative bacterium, by the well diffusion method. Mueller- Hinton Agar (MHA) was used as a medium, and 1 mg/mL of the samples were prepared to test the antibacterial activity. The MHA plates were swabbed with a test organism (OD equivalent to 0.5 McFarland). Each prepared sample solution (50 μL) was poured into different wells and incubated for 24 h at 37 °C. After incubation, the zone of inhibition was measured and expressed in mm. Standard antibiotics were included as positive controls in the antibacterial assay to validate the experimental results. Chloramphenicol was used as the positive control against *Staphylococcus aureus*, while norfloxacin was employed as the positive control against *Escherichia coli*. These antibiotics were selected based on their well-established antibacterial efficacy against Gram-positive and Gram-negative bacteria, respectively.

### 2.4 *In-vitro* antioxidant activity

The antioxidant property of ZIF-67, Mn doped ZIF-67, Zinc doped ZIF-67 and Mn/Zn codoped ZIF-67 nanostructures was assessed by evaluating their DPPH free radical scavenging capacity using the earlier reported method [32]. Briefly, 0.28 mL of DPPH solution (0.1 mM in 50% methanol) was mixed with different volumes of ZIF-67 based nanostructures solution (0.2–0.10 mL) and the final volume of the solution was made up to 2 mL with methanol (50%). The reaction mixture was then allowed to stand at 37 °C for 45 min. The absorbance of the solution was measured at 520 nm in a spectrophotometer using methanol (50%) and methanolic DPPH as blank solution and control, respectively. From the absorbance, the radical scavenging percentage (% RS) of Z, ZZ, ZM and ZMZ nanostructures was calculated using Eq. (1).

(1) $$\%\;\text{RS}\;=\frac{A_{control-A_{MOF/standard}}}{A_{Control}}\;\times \;100$$

where A_control_ and A_MOF_ /standard for the absorbance of control and Z, ZZ, ZM, ZMZ MOFs / ascorbic acid, respectively. The experiments were carried out in triplicates, and the DPPH radical-scavenging percentage is reported as the mean with the standard deviation. Ascorbic acid was used as a standard.

### 2.5 *In-vitro* cytotoxicity study

#### 2.5.1 Cell culture conditions

Human lung adenocarcinoma cells (A549) were cultured in DMEM supplemented with 10% FBS and 1% antibiotic-antimycotic solution. Cells were maintained at 37 °C and 5% CO_2_ in a humidified atmosphere. They were sub-cultured using trypsinization protocol once they attained 70% confluence and used for the experiments after three consecutive passages.

#### 2.5.2 Evaluation of cytotoxicity using methyl thiazolyl tetrazolium (MTT) assay

A549 cells (5000 cells/well) were seeded into 96-well microtiter plates and incubated at 37 °C and 5% CO_2_. After adherence, they were treated with different concentrations of the test compounds and further incubated for 48 h. Post-treatment, cells were added to the MTT reagent (1 mg/mL) and incubated for an additional 4 h. The formed formazan crystals were solubilised with DMSO, and absorbance was recorded using a multimode microplate reader at 570 nm (FluoSTAR Omega, BMG Labtech). Percentage cytotoxicity was calculated with respect to the untreated cell control.

#### 2.5.3 Apoptosis detection using acridine orange-ethidium bromide (AO-EB) dual fluorescence staining

A549 cells were seeded at a density of 20,000 cells/well in 24-well plates in complete DMEM and incubated at 37 °C and 5% CO_2_. Cells were added with the test compounds at the IC50 concentrations and further incubated for 48 h, as described previously. Cells were stained using acridine orange-ethidium bromide (AO-EB, 1:1) at 37 °C under dark for 20 min. Staining solution was removed, excess stain was washed using 1X PBS and cells were overlaid with PBS. Cells were imaged using a fluorescence imager (Zoe, BioRad) at 20X magnification under the green and red channels.

#### 2.5.4 Nuclear damage detection using hoechst staining

A549 cells were seeded onto 24-well plates at a density of 20,000 cells/well in complete DMEM and incubated at 37 °C and 5% CO_2_ conditions. Cells were treated with the test compounds at the optimum concentrations and further incubated for 48 h. After treatment, spent media was removed; cells were stained using Hoechst solution (5 µg/mL) for 15 min in dark at 37 °C. Staining solution was removed, excess stain was washed using 1X PBS and cells were overlaid with PBS. Cells were imaged using a fluorescence imager (Zoe, BioRad) at 20X magnification under the blue channel.

#### 2.5.5 Cytocompatibility testing on normal lung epithelial cells

BEAS-2B bronchial epithelial cells were seeded onto 96 well microtiter plates in complete DMEM and incubated at 37 °C and 5% CO_2_ conditions. Post-adherence, they were treated with different concentrations of the MOFs (50–250 µg/mL) and incubated further for 48 h. Cytotoxicity of the compounds was determined using the MTT assay, as described previously under 2.5.2.

#### 2.5.6 Evaluation of apoptotic protein expression by western blotting

A549 cells were seeded in 100 mm culture dishes and allowed to adhere overnight before treatment with the selected MOFs at their respective IC₅₀ concentrations for 48 h. Untreated cells served as the control. Following treatment, cells were washed with ice-cold PBS and lysed using RIPA buffer containing protease inhibitors. Total protein was collected after centrifugation, and protein concentration was determined using the Bradford assay. Equal amounts of protein (30 μg) were separated by 10% SDS-PAGE and transferred onto PVDF membranes. Membranes were blocked with 5% non-fat milk in TBST for 1 h and incubated overnight at 4 °C with primary antibodies against Bax, Bcl-2, Cleaved caspase-3, and β-actin (loading control). After washing, membranes were incubated with HRP-conjugated secondary antibodies for 1 h at room temperature. Protein bands were detected using an enhanced chemiluminescence (ECL) system, and band intensities were quantified using ImageJ software.

### 2.6 Material characterization

X-ray diffraction pattern of synthesized ZIF-67 samples were recorded on a Rigaku D/max-2500 advanced eco equipment between 5° and 90°. Morphological and elemental analysis were obtained using Scanning electron microscope ZEISS EVO 18 special edition coupled with Energy Dispersive Spectroscopy (EDS). The functional groups of the samples were examined using Fourier transform infrared spectroscopy (FTIR), with measurements conducted at room temperature on a Perkin Elmer Spectrum FTIR spectrometer. The spectra were recorded between 4000 and 450 cm⁻¹ with a resolution of 4 cm⁻¹. Thermal gravimetric analysis (TGA) measurements for all samples were conducted using a PerkinElmer TGA 4000 instrument. The measurements were performed under nitrogen atmosphere over a temperature range from 30 °C to 700 °C at a heating rate of 10 °C min⁻¹. Specific surface area (S_BET_) and porosity (total pore volume and pore radius) were measured using the Bellsorp gas sorption analyser based on the Brunauer-Emmett-Teller (BET) and Barret-Joyner-Halenda (BJH) models, respectively. The XPS analysis was performed using a Thermofisher Nexsa XPS instrument with source Al k alpha and Vacuum 10^−8^ mbar with a dwell time of 10 ms. Dynamic Light Scattering measurements were performed using a NanoZS zetasizer system (Malvern Instruments) using water as dispersion medium. Measurement parameters were as follows: a laser wavelength of 633 nm (He–Ne), a scattering angle of 173° (fixed—without changing possibility), a measurement temperature of 25 °C, a medium viscosity of 0.8872 mPa⋅s, a medium refractive index of 1.330, and a material refractive index of 1.04. ICP-OES on a Thermo Fisher iCAPPRO series instrument.

## 3 Results

### 3.1 X-Ray diffraction studies

The X-ray diffraction (XRD) patterns of pristine ZIF-67 MOF (Z) and its doped derivatives (ZM, ZZ, and ZMZ) are presented in Fig. 2(a). The characteristic diffraction peaks of ZIF-67 appearing at 2θ ≈ 7.4°, 10.4°, 12.7°, 14.8°, 16.5°, 18.0°,22.1°, 24.5°,25.6°, 26.7°,29.6°,30.6°,31.6° and 32.5° correspond to the (011), (002), (112), (022), (013), (222), (114), (233), (224),(134), (044), (334), (244) and (235) planes, respectively, indicating the formation of a highly crystalline sodalite-type MOF framework (JCPDS No. 00-062-1030). Both undoped and doped samples show strong diffraction peaks almost at the same locations, and each peak closely fits the predicted pattern of pure ZIF-67 which suggests that all of the samples are pure phases. The diffraction patterns of the ZM, ZZ, and ZMZ derivatives exhibit nearly identical peak locations to the pristine ZIF-67, with no extraneous signals indicating the absence of phase impurities. The XRD patterns showed no additional diffraction peaks attributable to crystalline ZnO or manganese oxide phases. Thus, no crystalline metal oxide phases were detected by XRD, suggesting that the Zn and Mn dopants were incorporated into the ZIF-67 framework without the formation of detectable crystalline oxide impurities, maintaining the crystalline integrity.

**Fig. 2:**
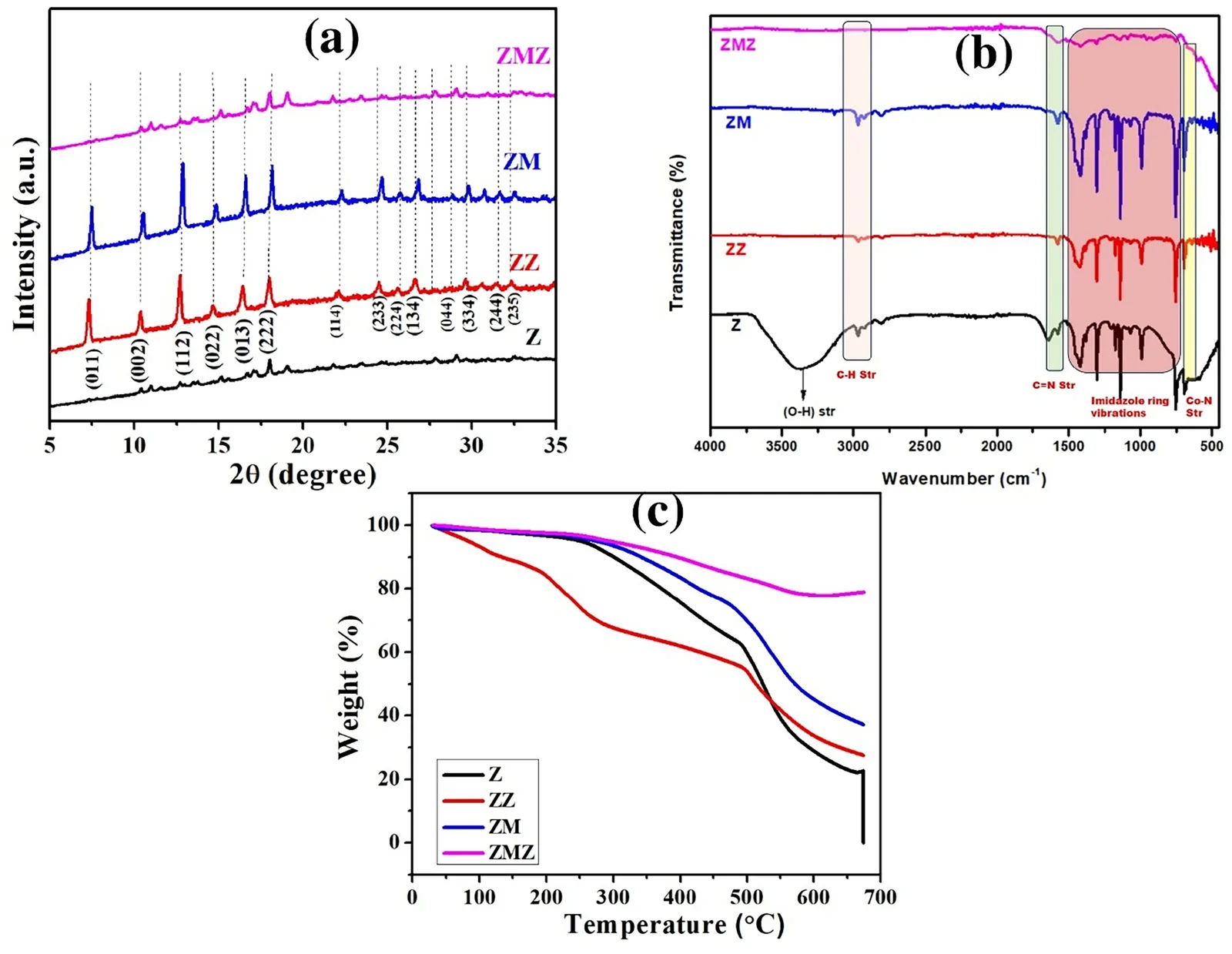
(a) XRD (b) FTIR (c) Thermogravimetric plot of (Z) ZIF-67, (ZZ) Zn-doped ZIF-67, (ZM) Mn-doped ZIF-67, and (ZMZ) Zn/Mn co-doped ZIF-67 respectively.

### 3.2 FTIR studies

The FTIR spectra of pristine ZIF-67 and its metal-doped derivatives (ZM, ZZ, and ZMZ) are shown in Fig. 2(b). All samples exhibit characteristic absorption bands typical of cobalt-based zeolitic imidazolate frameworks, supporting the preservation of the ZIF-67 structure after doping. The FTIR spectra exhibited a broad absorption band centred near 3400 cm⁻¹, corresponding to the stretching vibration of hydroxyl groups associated with surface-adsorbed moisture. Signals observed between 3130 and 2900 cm⁻¹ were assigned to aromatic and aliphatic C–H stretching vibrations of the imidazolate ligand. Characteristic absorption bands located at approximately 1580 and 1420 cm⁻¹ were attributed to the C=N and C–N stretching modes of the imidazole framework. Additional bands within the 1140–995 cm⁻¹ region were associated with C–N stretching accompanied by in-plane deformation of the imidazole ring. A prominent absorption band detected between 680 and 420 cm⁻¹ corresponded to the metal–nitrogen (Co–N) stretching vibration. No additional absorption bands indicative of secondary phases or impurity species were detected in the corresponding spectra.

### 3.3 TGA studies

Thermogravimetric analysis (TGA) was systematically employed to probe the thermal stability and decomposition kinetics of pristine ZIF-67 (Z) and a series of its metal-doped derivatives: zinc-doped (ZZ), manganese-doped (ZM), and zinc-manganese co-doped (ZMZ) as shown in the Fig. 2(c). The analysis reveals that the nature of the incorporated metal cation plays a decisive role in modulating the thermal flexibility of the zeolitic imidazolate framework. The pristine framework served as a benchmark, exhibiting excellent thermal stability with a characteristic, sharp single-step decomposition profile initiating around 350 °C. This abrupt weight loss is typical of a well-defined, homogeneous crystalline structure undergoing concerted ligand pyrolysis. The figure clearly shows about 8–9% mass loss and increased stability of the parent MOF up to 300 °C, proving the increased stability. The progressive decomposition of the 2-methylimidazole linkers at elevated temperatures results in significant structural degradation, ultimately leading to the collapse of the framework at hhigher temperatures.

### 3.4 BET studies

The nitrogen adsorption–desorption isotherms of the synthesized materials, namely undoped Z, single-doped ZZ, ZM, and co-doped ZMZ represented in Figure S1, provide valuable insights into their pore structures and surface characteristics. Prior to analysis, all samples were degassed at 120 °C for 12 h to remove adsorbed moisture and gases. The undoped ZIF-67 (Z) exhibits a typical Type I adsorption isotherm, characterized by a sharp increase in nitrogen uptake at very low relative pressures followed by an early plateau, indicating a predominantly microporous framework with a high specific surface area. The BJH pore diameter of approximately 1.40 nm is consistent with the intrinsic microporous nature of the pristine material.

The Zn-doped sample (ZZ) displays a slightly modified adsorption profile while largely retaining the characteristic features of the parent framework. The BJH pore diameter of 1.32 nm, which is comparable to that of the pristine sample, suggests that Zn incorporation has only a minor influence on the pore dimensions. The observed adsorption behaviour indicates subtle changes in the pore environment without significantly altering the overall microporous character of the material. Similarly, the Mn-doped sample (ZM) shows gradual nitrogen uptake following the initial micropore filling. The BJH pore diameter of 1.58 nm remains within the microporous range, suggesting that Mn incorporation induces only slight modifications to the pore structure while largely preserving the intrinsic framework of ZIF-67.

In contrast, the co-doped sample (ZMZ) exhibits a Type IV adsorption isotherm with a noticeable hysteresis loop, and the corresponding BJH pore diameter of 4.817 nm falls within the mesoporous range. Compared with the single-doped samples, these observations suggest that simultaneous incorporation of Zn and Mn results in more pronounced changes in the pore architecture. Although the BET surface area and total pore volume decrease after co-doping, the increase in pore diameter together with the adsorption behaviour indicates the development of mesoporous features within the framework. These changes may be associated with localized structural rearrangement, slight defect formation, or partial pore widening induced by co-doping. Overall, the results suggest that co-doping may modify the pore size distribution while preserving the underlying porous framework.

### 3.5 Dynamic light scattering (DLS) and zeta potential analysis

DLS study was employed using water as the dispersion medium to compare the hydrodynamic size characteristics of pristine Z (ZIF-67), modified ZZ, ZM, and ZMZ samples as seen in Figure S3 to evaluate their suitability for anticancer applications. The pristine Z sample exhibited hydrodynamic diameter of 86.18 nm and a PDI of 0.293. The intensity distribution was primarily characterized by a major peak at 129.5 ± 76.3 nm with an intensity contribution of 96.3%**,** indicating that the sample predominantly consists of nanosized particles exhibiting acceptable dispersion characteristics. Upon surface modification in the ZZ formulation, a slight reduction in hydrodynamic diameter (∼85 nm) and improved size homogeneity (PDI ∼0.290) were observed, suggesting enhancement in colloidal stability characterized by a major peak at 127.6 ± 75.24 nm with an intensity contribution of 97.5%**.** The ZM sample showed the smallest average hydrodynamic size (∼76 nm) with a comparable PDI (0.289), reflecting effective size regulation following modification. The major peak showed 100% intensity at 125.4 ± 66.51. In contrast, ZMZ showed a marginal increase in hydrodynamic diameter (∼94 nm) and a higher PDI (0.37). It showed a major peak at 126.8 ± 81.39 indicating increased surface complexity and hydration.

The zeta potential measurements revealed noticeable differences in the surface charge characteristics of the pristine and metal-doped MOFs (Table S2). The undoped material exhibited a zeta potential of −13.43 ± 1.47 mV, indicating a moderately negative surface with limited electrostatic stabilization in aqueous suspension. Following metal incorporation, the surface charge changed considerably. The Zn-doped sample showed the most negative zeta potential (−44.00 ± 2.47 mV), while the Mn-doped material exhibited a value of −34.43 ± 1.58 mV. These observations suggest that introducing Zn or Mn into the framework altered the surface environment, leading to an increase in the net negative surface charge and stronger electrostatic repulsion between particles. Such changes may be generally associated with improved dispersion stability and reflect modifications in the surface coordination environment brought about by metal substitution.

Interestingly, the co-doped MOF displayed a zeta potential of −11.20 ± 1.59 mV, which was less negative than those of the singly doped samples and comparable to the pristine material. This behavior indicates that the combined incorporation of Zn and Mn influenced the surface properties differently from individual doping. Rather than producing an additive effect, the simultaneous presence of both dopants may have resulted in a redistribution of surface charge or changes in the arrangement of surface-active sites, yielding a lower net surface potential. Overall, the zeta potential results demonstrate that metal doping modifies the interfacial characteristics of the MOF, although the extent and direction of the change depend on the nature and combination of the incorporated metal ions.

### 3.6 FESEM/EDS analysis

Field Emission Scanning Electron Microscopy (FESEM) was employed to investigate the surface morphology and microstructural features of pristine ZIF-67 and its metal-doped derivatives as shown in Fig. 3. Low-magnification images of undoped ZIF-67 reveal a relatively uniform distribution of particles with well-defined polyhedral morphology, indicating successful framework formation. The particles appear densely packed with slight agglomeration, which is commonly observed in MOF-based materials due to high surface energy.

**Fig. 3:**
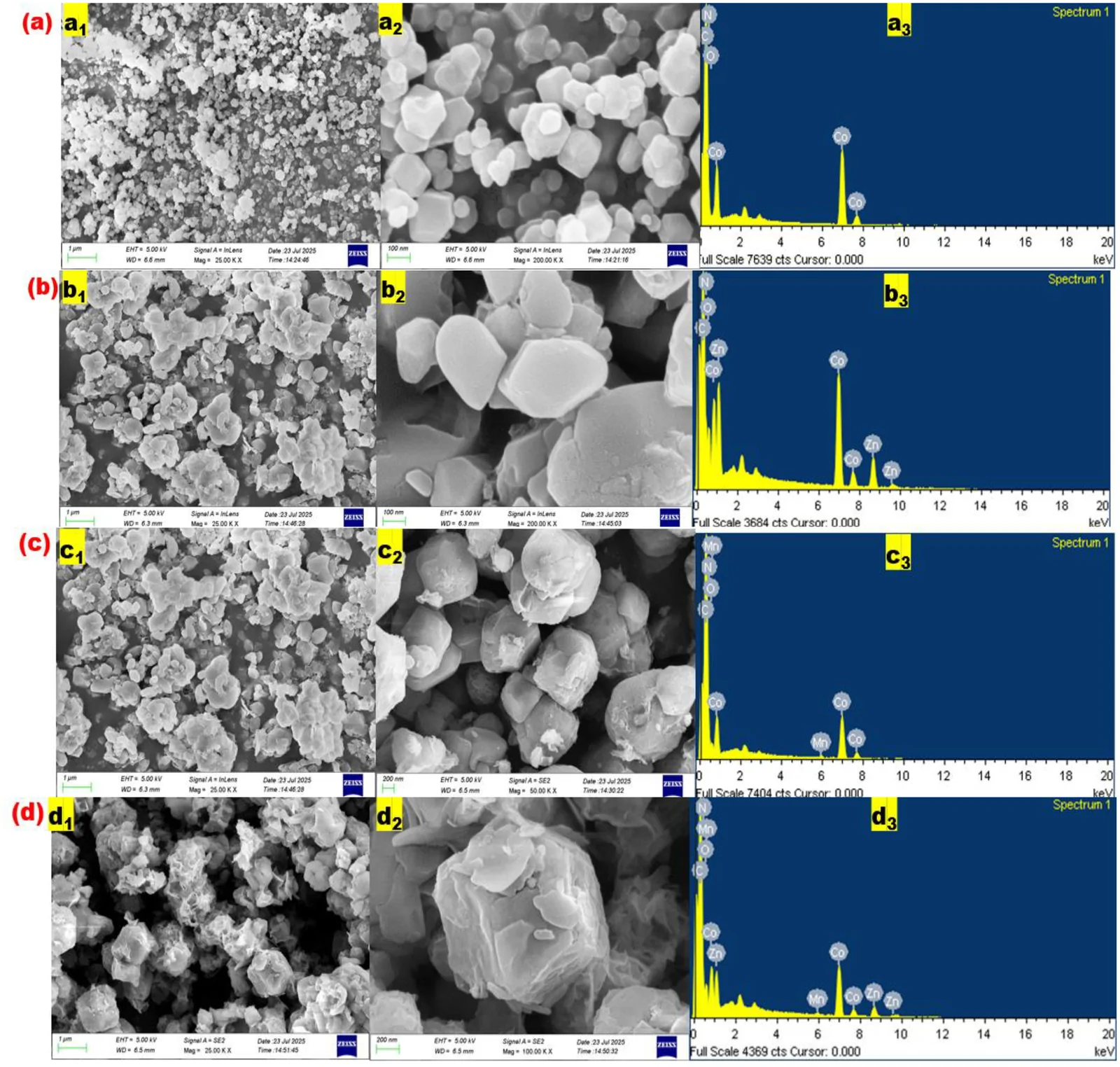
FESEM micrographs in low and high magnifications (a_1_-a_2_,b_1_-b_2_,c_1_-c_2_,d_1_-d_2_) and EDS spectra (a_3_,b_3_,c_3_,d_3_) (a) ZIF-67, (b) Zn-doped ZIF-67, (c) Mn-doped ZIF-67, and (d) Zn/Mn co-doped ZIF-67, respectively.

Upon Zn doping, noticeable changes in surface texture and particle size are observed. The Zn-doped ZIF-67 exhibits more compact and smoother polyhedral structures, suggesting that the introduction of Zn ions influences the crystal growth process and enhances structural regularity. In contrast, Mn-doped ZIF-67 displays comparatively rougher surfaces with irregularly shaped particles, indicating lattice distortion induced by Mn incorporation.

The Zn/Mn co-doped ZIF-67 sample shows a distinct morphological evolution characterized by enlarged particles with layered and stacked features. This altered morphology suggests the effect of dual-metal doping, which modifies nucleation kinetics and promotes anisotropic growth. High-magnification FESEM images further demonstrate the preservation of the overall ZIF-67 framework while highlighting surface roughness and edge irregularities introduced by dopant ions. Overall, the FESEM observations demonstrate that metal doping significantly affects particle morphology, surface roughness, and aggregation behaviour without fully collapsing the framework structure. These morphological modifications are expected to enhance active surface sites, which may positively influence the electrochemical and catalytic performance of the doped ZIF-67 materials.

Images (a_3_, b_3_, c_3_, d_3_) shown in the Fig. 3 represent the EDS analysis and the chemical composition of pure and doped ZIF-67 compounds. From the obtained spectra, percentage compositions of various elements, including Co, Zn, Mn, C, N and O are observed. The incorporation of Zn and Mn ions significantly affected the structural and biological properties, as indicated in the SEM micrographs. Additionally, the spectrum revealed that the synthesised samples contained no additional foreign elements. The percentage composition of the elements present as observed in EDS is displayed in Table S3.

### 3.7 Transmission electron microscopy (TEM) studies

Transmission electron microscopy images obtained at low and high magnifications represented in the Fig. 4 provide detailed insight into the nanoscale morphology of ZIF-67 and its Zn, Mn, and Zn/Mn-doped counterparts. It revealed that pristine ZIF-67 consists of uniformly distributed polyhedral nanoparticles with smooth surfaces and distinct particle boundaries, indicating the successful formation of a well-defined crystalline framework. Following zinc and manganese incorporation, noticeable changes in particle morphology were observed. The doped samples exhibited increased surface irregularity, slight variations in image contrast, and localized particle agglomeration in comparison to the pristine material. In the Zn/Mn co-doped ZIF-67 sample, these morphological changes became more evident, with fused particles, overlapping crystalline domains, and heterogeneous contrast distributed throughout the nanoparticles. Despite these structural modifications, dual-metal doping led to a noticeable disruption of the characteristic polyhedral morphology of the framework, as evidenced by the FESEM images. The average particle size analysis determined by TEM images is shown in the Figure S2. TEM particle-size distribution analysis revealed that pristine ZIF-67 possessed an average particle size of approximately 95.74 ± 42.32 nm, whereas the single-doped samples exhibited larger average particle sizes of 188.8 ± 90.51 and 181.8 ± 54.9 nm for ZZ and ZM respectively. In contrast, the dual-doped sample showed a broader particle-size distribution with an average size of 102.9 ± 22.86 nm, suggesting that simultaneous incorporation of both dopants may influence the particle nucleation and growth behaviour differently from individual doping.

**Fig. 4:**
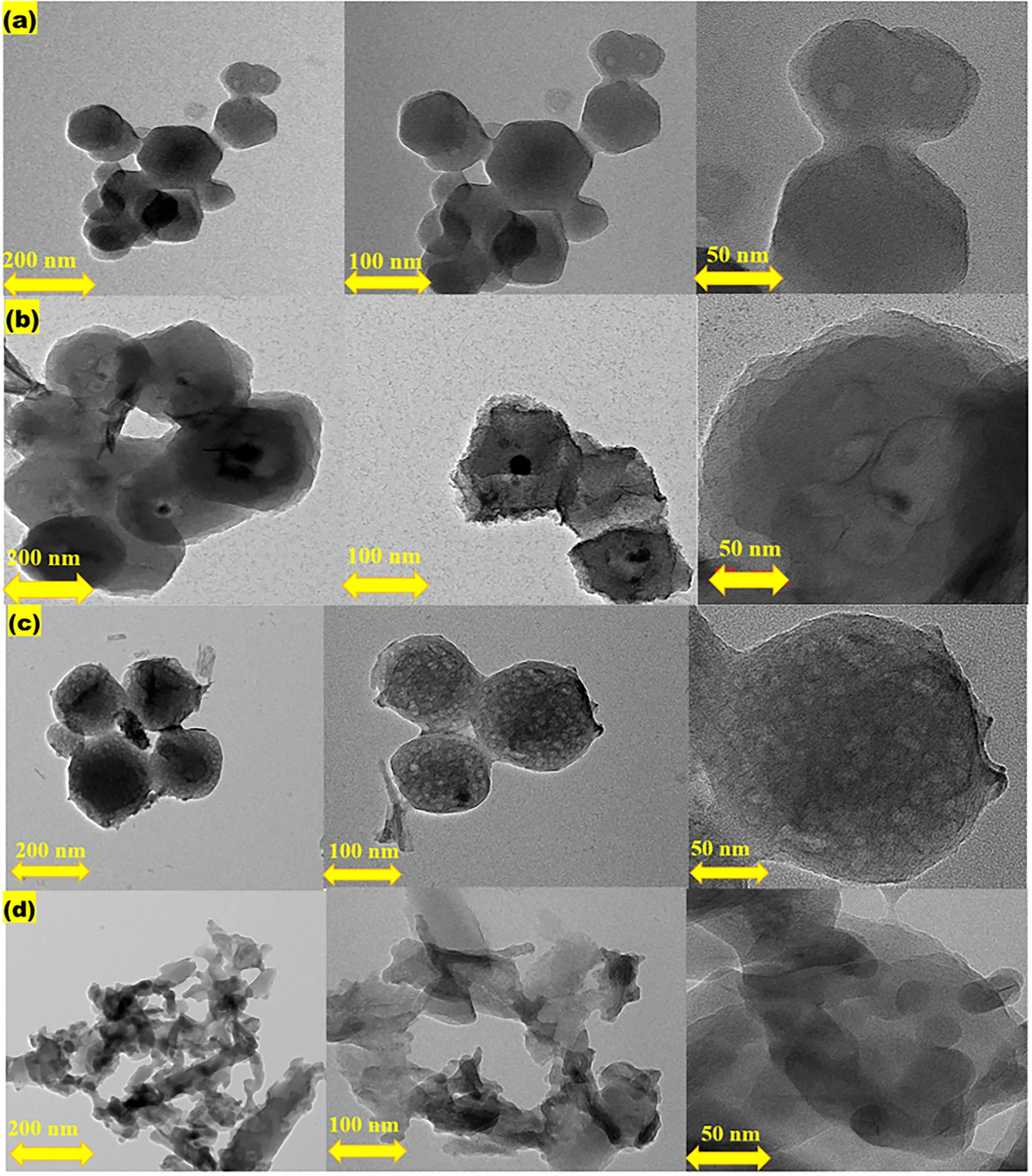
(i) Transmission electron micrographs in low and high magnifications of the samples (a) ZIF-67, (b) Zn-doped ZIF-67, (c) Mn-doped ZIF-67, and (d) Zn/Mn co-doped ZIF-67 respectively.

### 3.8 XPS studies

The surface elemental composition and chemical states of the synthesized Zn/Mn-modified ZIF-67 were investigated using surface sensitive X-ray photoelectron spectroscopy (XPS) as seen in the Figure S4. The survey spectrum suggested the presence of C, N, O, Co, Zn, and Mn, demonstrating the incorporation of the dopant metals into the framework. The high-resolution C 1s spectrum exhibited characteristic contributions corresponding to C–C/C=C, C–N, and N–C=N bonds associated with the 2-methylimidazole linker. Compared with pristine ZIF-67, slight shifts in the binding energies of carbon species were observed after metal incorporation, indicating modifications in the electronic environment of the framework. The N 1s spectrum was deconvoluted into components attributed to pyridinic nitrogen and metal-coordinated nitrogen species (M–N), indicating the preservation of the coordination network while suggesting charge redistribution resulting from Zn and Mn incorporation. Furthermore, the O 1s spectrum could be resolved into peaks associated with lattice oxygen/metal–oxygen bonds and surface hydroxyl or adsorbed oxygen-containing species, indicating the presence of oxygenated surface functionalities and partial metal–oxygen interactions generated during synthesis and post-treatment.

The Co 2p spectrum displayed prominent Co 2p₃/₂ and Co 2p₁/₂ peaks accompanied by characteristic shake-up satellite features, substantiating that cobalt predominantly exists in the Co²⁺ oxidation state within the ZIF framework. A slight positive shift in the Co binding energies relative to pristine ZIF-67 suggests electron transfer between cobalt centres and the incorporated dopant metals, leading to a more electron-deficient cobalt environment. The Zn 2p spectrum exhibited well-defined Zn 2p₃/₂ and Zn 2p₁/₂ peaks centred near 1021–1023 eV and 1044–1046 eV, respectively, characteristic of Zn²⁺ species. The absence of metallic Zn signals indicates that zinc is fully oxidized and maybe integrated into the framework through coordination with nitrogen-containing ligands. The Mn 2p spectrum showed Mn 2p₃/₂ and Mn 2p₁/₂ peaks within the expected binding-energy range for oxidized manganese species. Detailed peak analysis suggests the coexistence of Mn²⁺ and Mn³⁺ states, reflecting the variable-valence nature of manganese and its strong interaction with the surrounding coordination environment. The coexistence of multiple manganese oxidation states may create additional active sites and promote electron-transfer processes within the framework. Collectively, the observed shifts in Co, Zn, and Mn binding energies, together with the presence of metal–nitrogen and metal–oxygen interactions, suggest successful metal incorporation and reveal significant modulation of the surface electronic structure. These changes are expected to contribute to enhanced physicochemical properties and improved performance in catalytic, electrochemical, and biomedical applications.

### 3.9 ICP-OES studies

The elemental composition of pure ZIF-67 and the Zn/Mn co-doped ZIF-67 was quantitatively determined using inductively coupled plasma-optical emission spectroscopy (ICP-OES). Ten milligrams of each sample were fully digested in ICP-grade nitric acid and diluted in accordance with the protocol outlined in the experimental section prior to analysis. Excellent linearity (R2 > 0.997) was shown in the calibration curves for Co, Zn, and Mn, demonstrating the validity of the quantitative analysis across the concentration range used.

The ICP-OES results are presented in Table S4. Pristine ZIF-67 exhibited a cobalt concentration of 4.655 ± 0.06 ppm, whereas the co-doped material contained 4.441 ± 0.05 ppm Co, 0.161 ± 0.003 ppm Zn, and 0.573 ± 0.007 ppm Mn. The slight reduction in cobalt concentration following the introduction of Zn and Mn suggests that a fraction of the framework Co²⁺ ions was replaced during synthesis while maintaining cobalt as the predominant metal species within the framework.

### 3.10 Biological studies

The *in vitro* antibacterial activity of the samples Z, ZM, ZZ, and ZMZ was evaluated against *Staphylococcus aureus* and *Escherichia coli* using the agar well diffusion method. The antibacterial assay was performed using Mueller–Hinton agar, with the bacterial inoculum adjusted to 0.5 McFarland standard. Sample solutions were prepared at a concentration of 1 mg/mL, and 50 μL of each sample was introduced into the respective wells. The inoculated plates were incubated at 37 °C for 24 h, following which the zones of inhibition were measured and expressed in millimetres. Chloramphenicol and norfloxacin were used as positive controls against *S. aureus* and *E. coli*, respectively. The antibacterial activity of the synthesized samples was evaluated in triplicates by comparing the measured zones of inhibition with those obtained for the respective positive controls. ZZ, ZM, and ZMZ showed zone of inhibition against *Staphylococcus aureus* measuring 8 ± 0.2 mm, 14 ± 0.4 mm and 16 ± 0.3 mm, respectively while Z showed no change .With *E. coli,* samples Z, ZZ, ZM, and ZMZ showed an inhibition zone of 9 ± 0.2 mm, 9 ± 0.3 mm, 11 ± 0.2mm and 15 ± 0.1 mm, respectively as shown in Figure S5. The minimum inhibitory concentration (MIC) of the samples was further determined using the broth microdilution method. The samples were tested at concentrations of 1000, 500, 250, 125, 62.5, 31.25, and 15.63 μg/mL against both bacterial strains. Following incubation at 37 °C for 24 h, resazurin dye was added as an indicator of bacterial metabolic activity. Absorbance was subsequently measured at 570 nm using a microtiter plate reader. The MIC was recorded as the lowest concentration at which bacterial metabolic activity was inhibited.

The minimum bactericidal concentration (MBC) was determined by subculturing aliquots from the respective sample concentrations onto agar plates before the addition of resazurin dye. After incubation at 37 °C for 24 h, the lowest concentration showing no visible bacterial growth was recorded as the MBC. The antibacterial evaluation demonstrates significant strain-specific bioactivity among the synthesized formulations, with notable effectiveness against the Gram-negative pathogen *Escherichia coli*. The unmodified formulation, Sample Z, exhibited superior antibacterial efficacy, registering the lowest Minimum Inhibitory Concentration (MIC) of 31.25 µg/mL and the lowest Minimum Bactericidal Concentration (MBC) of 250 µg/mL against *E. coli*. These metrics confirm Sample Z as the most potent candidate within the experimental series for Gram-negative bacterial suppression. Strategic structural modifications further enhanced the inhibitory profiles against the Gram-positive strain, *Staphylococcus aureus*. Specifically, formulations ZM and ZMZ demonstrated a two-fold enhancement in growth inhibition compared to the baseline material, successfully reducing the MIC threshold from 500 µg/mL to 250 µg/mL. These findings validate Sample Z as a highly promising lead material for Gram-negative microbial control, while establishing a viable foundation for future structural optimization aimed at maximizing broad-spectrum bactericidal performance. The MIC/MBC results of the samples corresponding to both the organisms are tabulated in Table S6.

The free radical scavenging activity of the synthesised samples (Z, ZZ, ZM, and ZMZ) was systematically evaluated in triplicates across increasing concentrations (200–1000 µg/mL) and benchmarked against the standard antioxidant, ascorbic acid (Figure S7). As expected, ascorbic acid exhibited consistently high scavenging efficiency (>90%) at all tested concentrations, confirming its well-established role as a potent reference antioxidant. In contrast, all synthesized materials exhibited moderate but clearly distinguishable antioxidant activity, with a concentration-dependent variation in scavenging efficiency characteristic of heterogeneous, surface-mediated antioxidant systems. Among the synthesized samples, ZMZ showed the highest free radical scavenging activity at all concentrations, followed by ZM, Z, and ZZ. A gradual increase in radical scavenging was observed from 200 to 1000 µg/mL for all materials, suggesting a concentration-dependent response. Among the tested samples, the co-doped ZMZ material consistently showed the highest scavenging activity, reaching approximately 52% at 1000 µg/mL. The pristine ZIF-67 (Z) displayed a relatively stable scavenging efficiency of around 33–41%. The Mn-doped sample (ZM) exhibited a modest increase in activity with concentration, attaining approximately 42% at the highest concentration. In contrast, the Zn-doped sample (ZZ) showed comparatively lower radical scavenging throughout the concentration range, although a gradual improvement was evident at higher concentrations.

The cytotoxic potential of synthesized metal-organic framework (MOF) samples, including Z (undoped), ZZ, ZM, and ZMZ (co-doped), was evaluated using the MTT assay on cultured human cells. The results indicate a concentration-dependent cytotoxicity profile for all samples. The undoped sample Z exhibited moderate cytotoxicity, with 17.8% cell death at 50 µg/mL, increasing to 86.6% at 250 µg/mL, and an IC_50_ value of 130.24 µg/mL. The ZZ sample showed a slightly higher IC_50_ of 132.97 µg/mL, suggesting a comparable but marginally less potent cytotoxic effect at lower concentrations. Interestingly, the ZM sample displayed an IC_50_ of 123.11 µg/mL, indicating that the single dopant enhanced cytotoxicity slightly at intermediate concentrations. The co-doped ZMZ sample exhibited the most pronounced cytotoxicity even at lower concentration, with an IC_50_ of 94.89 µg/mL, demonstrating that co-doping significantly increased cytotoxic potential compared to the other samples.

The acridine orange/ethidium bromide (AO/EB) fluorescence staining method was used to evaluate the degree of membrane integrity loss and apoptosis caused by the undoped (Z) and doped (ZZ, ZM, and ZMZ) as shown in Fig. 5(a). Among the studied materials, the ZMZ codoped sample exhibited the more pronounced cytotoxic profile. The fluorescence images showed total loss of membrane integrity and extensive EB inclusion, with strong red emission predominating. Strong orange-red overlays in the merged picture indicated widespread late-stage apoptosis and cell death. Cells treated with the undoped Z (ZIF-67) sample displayed mild but notable nuclear alterations compared to the control. Although many nuclei retained their typical morphology, several cells showed slight hyper fluorescence and early chromatin condensation. These features suggest that Z induces low-level nuclear stress and initiates apoptotic signalling in a limited fraction of cells. Importantly, the nuclear changes remained modest, indicating that undoped ZIF-67 possesses only moderate inherent cytotoxic potential as depicted by MTT assay. More pronounced nuclear alterations were observed in cells treated with the ZZ and ZM doped samples. In these groups, hoechst staining revealed a significant increase in fluorescence intensity, reflecting chromatin condensation typical of apoptotic nuclei. The ZMZ co-doped sample had the most pronounced effect on nuclear morphology. Hoechst-stained nuclei in this treatment group exhibited extremely bright fluorescence, extensive condensation, and widespread fragmentation, with several cells showing classical apoptotic bodies and severely distorted nuclear shapes.

**Fig. 5:**
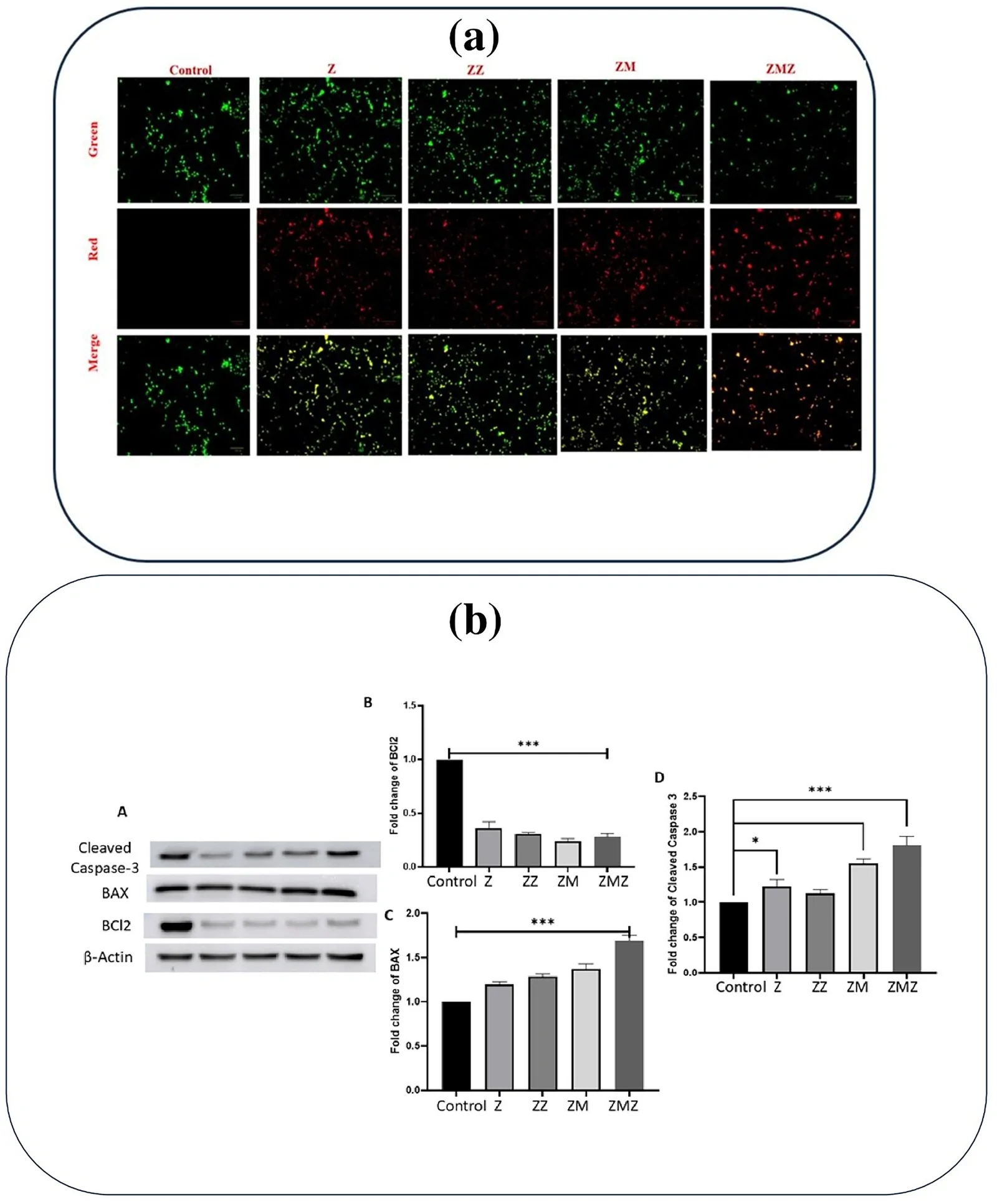
(a). Fluorescence staining images of (Z) ZIF-67, (ZM) Mn-doped ZIF-67, (ZZ) Zn-doped ZIF-67, (ZMZ) Zn/Mn co-doped ZIF-67, respectively. 5 (b): Protein expression analysis of A549 cells treated with the MOFs. (A) Representative blots from different treatment groups, Densitometric analysis of (B) BCl2 (C) Bax and (D) Cleaved caspase-3.*p < 0.05 and ***p < 0.001 compared to control.

Western blot analysis as shown in Fig. 5(b) was performed to evaluate the expression of apoptosis-related proteins following treatment with the synthesized MOFs. Densitometric quantification, normalized against β-actin, showed significant differences in protein expression compared with the untreated control. Cleaved caspase-3 expression decreased after treatment, while BAX expression increased in all treated groups, with the highest level observed for the Zn, Mn-codoped ZIF-67 (ZMZ) sample. In contrast, Bcl-2 expression was markedly reduced following treatment. Statistical analysis confirmed that these changes in protein expression were significant among the experimental groups (*p* < 0.05).

## 4 Discussion

### 4.1 Material characterization

The consistent peak positions in XRD across the pristine and single doped samples provide evidence of the framework inherent structural stability despite chemical modification. Since Mn²⁺ and Zn²⁺ can occupy or partially substitute the Co²⁺ sites in the ZIF-67 structure, their incorporation is not necessarily expected to generate new independent diffraction peaks in ZM/ZZ or ZMZ. Instead, doping may be reflected by subtle changes in peak position, intensity, or peak broadening arising from variations in the local coordination environment and lattice parameters. Thus, the preservation of the characteristic ZIF-67 diffraction pattern, together with the absence of extra crystalline reflections, may support the successful incorporation of Mn and Zn without detectable secondary crystalline phases as evidenced in the literature[33,34]. The observed reduction in diffraction peak intensity, particularly for the ZMZ sample, indicates a partial loss of long-range structural order. This decrease may arise from local lattice distortion and structural disorder associated with the incorporation of Zn²⁺ and Mn²⁺ ions into the ZIF-67 framework, which can perturb the coordination environment of the parent Co²⁺ sites [35,36]. Furthermore, the absence of crystalline impurities depict that these ions may be substituted into the Co-N₄ coordination environments of the framework rather than forming separate mineral phases. Previous studies on ZIF-67 have demonstrated that structural modifications resulting from the incorporation of secondary metal ions can be investigated through rietveld refinement and lattice parameter analysis. Such studies have shown that metal incorporation may produce subtle changes in the unit-cell parameters and diffraction peak positions while maintaining the fundamental ZIF-67 crystal framework [37]. The incorporation of Zn and Mn into the ZIF-67 framework is supported by complementary ICP-OES, XPS, EDS mapping, XRD, and TEM analyses. Although XPS is surface-sensitive and cannot independently confirm bulk incorporation, the combined compositional, elemental, and structural evidence supports Zn and Mn incorporation. Precise determination of their lattice positions and local coordination environments would require techniques such as XANES/EXAFS or reliable Rietveld refinement; however, the reduced crystallinity of the doped samples limits the applicability of Rietveld analysis in the present study.

The FTIR image explains that the observed vibrational features are characteristic of ZIF-67 framework and demonstrate successful coordination between Co²⁺ ions and the nitrogen atoms of the imidazolate linker, leading to the formation of the metal–organic network. The presence of the Co–N stretching vibration in the low-wavenumber region supports the establishment of coordination bonds essential for framework construction. Furthermore, the retention of the characteristic imidazole-related bands, together with the absence of unexpected absorption peaks, indicates that the incorporation of dopant ions did not alter the fundamental chemical structure of ZIF-67. In the doped samples (ZM, ZZ, and ZMZ), the main functional group positions remain largely unchanged, implying that Mn²⁺ and/or Zn²⁺ ions maybe incorporated into the ZIF-67 framework without disrupting its chemical bonding environment [38]. These findings suggest that the crystalline framework remained chemically intact following the doping process, consistent with previous reports on doped ZIF-based materials [39,40].

Thermal stability studies on the samples reveal that the introduction of manganese (ZM) into the ZIF-67 structure resulted in a modest and slight increase in thermal stability, as indicated by a slightly higher temperature of decomposition. This suggests that the substitution of Co²⁺ with Mn²⁺ introduces slightly less destabilisation, potentially due to minimal differences in the coordination geometry. In contrast, the incorporation of zinc (ZZ) had a profoundly more significant impact. The ZZ sample displayed a broadened decomposition profile occurring at a substantially lower temperature. This behaviour is likely a characteristic of a less robust and more heterogeneous framework. Fascinatingly, the co-doped material (ZMZ) exhibited a thermal profile intermediate between those of ZM and pristine ZIF-67 [41].While the destabilising influence of zinc remains evident, its effect is mitigated relative to the solely zinc-doped sample. This indicates that the co-presence of manganese and zinc creates a distinct coordination environment, in which the frameworks overall thermal stability is a composite result of the competing influences of the two dopant ions. In conclusion, this study unequivocally demonstrates that the thermal degradation pathway of ZIF-67 can be precisely tailored through selective metal doping [42]. The choice of dopant not only alters the chemical composition but also directly governs the frameworks thermal robustness.

The doped samples displayed a decrease in BET surface area along with modifications in particle size and shape when compared to pristine ZIF-67. On incorporation of Zn and Mn to the ZIF-67 framework, the synthetic materials' morphological and textural characteristics showed a methodical evolution. The structural alterations shown in the XRD patterns can be directly linked to these modifications. In contrast to the Zn- and Mn-doped samples, which showed decreased peak intensity and slight peak broadening due to lattice distortion likely caused by the substitution of Co²⁺ with Zn²⁺ and Mn²⁺ ions, pristine ZIF-67 displayed well-defined diffraction peaks typical of its highly crystalline sodalite framework. In the Zn, Mn-codoped ZIF-67 sample, structural distortion is more noticeable since the diffraction peaks are considerably weaker and appear broader, indicating partial amorphization [43]. The reduced crystallinity observed by XRD agrees with the TEM results, which show disruption of the well-defined polyhedral morphology of pristine ZIF-67 following Zn²⁺/Mn²⁺ co-doping. This structural distortion can reduce pore accessibility and account for the lower BET surface area and pore volume. The decrease may arise from altered crystal growth, increased structural disorder, and partial occupation or obstruction of microporous regions by the introduced metal ions. Similar reductions in surface area have been reported for bimetallic ZIF-67 systems. Overall, the combined XRD and TEM results suggest that the decrease in porosity is primarily associated with partial disruption of the porous framework rather than simple pore blocking. Although co-doping reduces structural order and accessible porosity, the resulting defect-rich framework may provide additional chemically active sites and facilitate metal-ion release, potentially contributing to the enhanced biological activity of the Zn/Mn-codoped ZIF-67 sample [43,44].

The BJH insets of the respective isotherms in BET, depict the pore size distributions, reinforce these observations: Z exhibits narrow micropores, ZZ and ZM display a broadened distribution retaining the microporosity, and ZMZ reveals a mesoporous network, indicating the successful tuning of porosity through doping strategies [45]. The various BET and BJH parameters are shown in Table S1 for ZIF-67 and its doped counterparts.

Low-magnification scanning electron microscopic images of undoped ZIF-67 reveal a relatively uniform distribution of particles with well-defined polyhedral morphology, indicating successful framework formation. The particles appear densely packed with slight agglomeration, which is commonly observed in MOF-based materials due to high surface energy [46]. The Zn-doped ZIF-67 exhibits more compact and smoother polyhedral structures, suggesting that the introduction of Zn ions influences the crystal growth process and enhances structural regularity. In contrast, Mn-doped ZIF-67 displays comparatively rougher surfaces with irregularly shaped particles, indicating slight lattice distortion induced by Mn incorporation. The Zn/Mn co-doped ZIF-67 sample shows a distinct morphological evolution characterized by enlarged particles with layered and stacked features.High-magnification FESEM images further indicate the preservation of the overall ZIF-67 framework while highlighting surface roughness and edge irregularities introduced by dopant ions. FESEM analysis indicates that metal doping modifies the morphology, surface roughness, and aggregation behavior of ZIF-67, with partial framework distortion observed in the Zn,Mn-codoped sample. These structural changes are expected to increase the availability of active surface sites, thereby enhancing the materials function.

TEM morphology of pristine ZIF-67 suggests the formation of a well-defined and ordered framework in the pristine structure, which has previously been associated with favourable characteristics for cellular uptake owing to its nanoscale dimensions. The morphological variations observed after Zn and Mn incorporation may indicate localized lattice distortion arising from the substitution of cobalt by dopant ions within the framework. These structural modifications could increase the number of exposed surface sites and alter the surface properties of the material, potentially influencing its interaction with biological membranes and intracellular components [47,48]. The greater structural heterogeneity observed in the Zn/Mn co-doped ZIF-67, including particle fusion, overlapping domains, and contrast variations, may reflect a higher degree of defect formation within the framework. Nevertheless, the preservation of the overall polyhedral framework suggests that the structural integrity of the MOF remains intact following metal incorporation, which may be advantageous for maintaining its functionality in biological environments [49].

The DLS measurements conducted on undoped, monodoped and dual doped samples demonstrated intensity-weighted distributions dominated by a single principal peak, showing the absence of significant aggregation and good dispersion quality. The synthesized MOFs possessed particle sizes below 100 nm along with a relatively narrow size distribution characteristics that have been associated in previous studies with improved cellular uptake and the potential for passive accumulation in tumour tissues through the enhanced permeability and retention (EPR) phenomenon. Although these features suggest possible suitability for anticancer drug delivery, the current work was confined to *in vitro* cytotoxicity assessment. Consequently, no *in vivo* investigations were undertaken to confirm whether these materials exhibit EPR-mediated tumour localisation under physiological conditions[50].

The survey XPS spectrum demonstrated the presence of Co, Zn, Mn, C, N, and O, indicating the coexistence of the parent ZIF-67 framework elements and the introduced metal species. The maximum binding energy of carbon’s C 1s photoelectron line was used as a charge reference, which was positioned at 284.8 eV [51,52]. All ZIF-based materials exhibited a symmetric N 1s photoelectron peak, centred at approximately 398.9 eV, which is attributed to the nitrogen atoms present in the organic linker [53]. The Co 2p spectrum is expected to exhibit two principal spin–orbit components corresponding to Co 2p₃/₂ and Co 2p₁/₂, accompanied by characteristic satellite features. The observed binding energies are consistent with cobalt predominantly existing in the +2-oxidation state, which is the expected coordination environment for cobalt ions within the ZIF-67 framework [54]. The observed separation of approximately 15 eV between these components is characteristic of cobalt in the material [55,56]. Minor shifts in the peak positions after Zn and Mn incorporation may reflect changes in the local electronic environment surrounding the cobalt centres rather than a complete change in their oxidation state. Such variations are likely associated with partial substitution of cobalt ions and electronic interactions between the dopant species and the imidazolate framework. The Zn 2p region for both samples shows the characteristic spin–orbit doublet, with the Zn 2p₃/₂ component appearing at approximately 1021 eV, consistent with Zn²⁺ species [57]. The absence of additional components attributable to metallic zinc or higher oxidation states would suggest that zinc is incorporated into the framework as divalent Zn²⁺ ions. Small changes in the binding energy relative to pristine materials may indicate differences in the coordination environment caused by interaction with neighbouring cobalt and manganese atoms. This supports the incorporation of zinc into the MOF lattice rather than the formation of separate zinc-containing phases [58]. Similarly, the deconvoluted Mn 2p spectrum revealed contributions from two manganese oxidation states. The components centred at approximately 641.0–641.2 eV and 652.8–653.0 eV were assigned to the Mn²⁺ 2p₃/₂ and Mn²⁺ 2p₁/₂ levels, whereas the components appearing at relatively higher binding energies, around 653.5–654.2 eV, were associated with Mn³⁺. These results indicate the coexistence of Mn²⁺ and Mn³⁺ species at the surface of the material. Such mixed-valence behaviour may arise from differences in the local coordination and electronic environment of Mn after its incorporation into the ZIF-67 framework [59,60]. The coexistence of Co²⁺, Zn²⁺ and Mn²⁺ suggests that metal substitution occurs without significant disruption of the fundamental coordination chemistry of the ZIF-67 framework.

The XPS analysis suggests the coexistence of Mn²⁺ and Mn³⁺ species in the Mn-containing ZIF-67 samples. The incorporation of Zn and Mn may modify the local coordination and electronic environment of the Co-based framework. Considering the nominally divalent states of Zn²⁺, Mn²⁺ and Co²⁺, their substitution at Co-associated sites may be represented in Kröger–Vink notation by Zn_Co_^×^​ and Mn_Co_^×^​, respectively [28]. The possible presence of Mn³⁺ can additionally be represented as Mn_Co_^∙^​, corresponding to Mn³⁺ occupying a site associated with Co²⁺. The coexistence of these species may result in local variations in charge distribution and coordination environment, which could contribute to the structural and morphological modifications observed after doping is represented in Eqs. (2), (3) and (4). These representations are proposed as a defect-chemical description of the observed XPS features and should not be interpreted as direct evidence of a specific atomic substitution site or charge-compensation mechanism [55,57,61].

(2) $$Co_{Co}^{x}\to Zn_{Co}^{x}$$

(3) $$Co_{Co}^{x}\to Mn_{Co}^{x}$$

(4) $$Co_{Co}^{x}\to Mn_{Co}^{.}$$

ICP-OES was employed to quantitatively determine the metal composition of pristine ZIF-67 (Z) and the Zn/Mn co-doped ZIF-67 (ZMZ). The elemental concentrations obtained from the analysis along with the standard deviation values are presented in Table S4. The pristine ZIF-67 sample contained cobalt at a concentration of 4.655 ppm, while zinc and manganese were not detected, indicating that the parent framework is composed exclusively of cobalt metal nodes while the co-doped material had 4.441 ppm Co, 0.161 ppm Zn, and 0.473 ppm Mn. The slight drop in cobalt content after doping is consistent with Zn²⁺ and Mn²⁺ partially replacing framework Co²⁺ ions while keeping cobalt as the main metal ingredient. When the elemental concentrations were converted to molar composition, the results were 87.19 mol% Co, 2.85 mol% Zn, and 9.96 mol% Mn. This resulted in a normalized metal ratio of Co_1.00_Zn_0.033_Mn_0.138_. The experimentally obtained composition shows preferential incorporation of Mn relative to Zn, despite the precursor solution being made with a Co: Zn: Mn molar ratio of 1:0.1:0.1. The ICP-OES results provide direct quantitative evidence for the formation of a multimetallic ZIF structure. These findings complement the XPS and EDS analyses, which verified the presence of Co, Zn, and Mn at the material surface, while ICP-OES suggests their bulk elemental composition. The combined characterization therefore supports the successful synthesis of the Zn/Mn co-doped ZIF-67 material and validates the incorporation of the dopant metals in the framework [[61], [62], [63]] ***.***

### 4.2 Biological studies

#### 4.2.1 Antibacterial study

*In vitro* antimicrobial activity of the samples Z, ZZ, ZM, ZMZ, was tested against *Staphylococcus aureus*, a Gram-positive bacterium, and *Escherichia coli*, a Gram-negative bacterium, by the Well diffusion method. The experiment was conducted in triplicates. The enhanced antibacterial activity of Zn/Mn-codoped ZIF-67 (ZMZ) compared with the singly doped materials (ZZ and ZM) can be attributed to the combined effect of Zn and Mn on the surface characteristics and local chemical environment of the ZIF-67 framework. The distinct zeta potentials observed for pristine ZIF-67 (−13.43 ± 1.47 mV), Zn-doped ZIF-67 (−44.00 ± 7.47 mV), Mn-doped ZIF-67 (−34.43 ± 1.58 mV), and ZMZ (−11.43 ± 1.57 mV) indicate that co-doping produces a different surface charge distribution from either individual dopant. This altered surface environment may strengthen interactions with bacterial cell membranes and facilitate interfacial processes such as metal-ion release and reactive species generation [64,65]. Thus, the improved bactericidal effect of ZMZ against both organisms is likely related to the synergistic influence of Zn and Mn on the surface and coordination environment, rather than simply the additive presence of two dopant metals [66,67]. Differences in the inhibition zones, MIC, and MBC values suggest variations in their growth-inhibitory and bactericidal effects. The observed activity may arise from interactions of the material and released metal ions with bacterial cells, leading to membrane disturbance, metabolic dysfunction, and oxidative stress. The different responses of the two bacteria can be attributed to variations in their cell-envelope structures. Overall, the results suggest that Zn and Mn incorporation modifies the antibacterial behaviour of the parent material and may enhance its effectiveness depending on the composition and bacterial strain. Thus, it is observed that the incorporation of Zn and Mn into ZIF-67 progressively perturbed the long-range structural order, accompanied by morphological alterations and a reduction in BET surface area, indicating changes in the framework architecture and accessible porosity. These structural modifications, together with the altered surface chemical states evidenced by XPS, can influence surface charge, metal-ion accessibility, and interactions with bacterial cell envelopes, thereby contributing to the enhanced antibacterial performance of the doped materials [[68], [69], [70]].

The comparison table for inhibition by using other related compounds for the same bacteria with reference to MIC/MBC concentration is shown in Table S7(a) and (b).

#### 4.2.2 Proposed mechanism of antibacterial activity

The antibacterial activity of the Zn/Mn co-doped MOF is proposed to arise from multiple complementary mechanisms involving interactions at the bacterial surface and subsequent intracellular damage as observed in the literature [[71], [72], [73]]. Initially, the MOF particles may interact with the bacterial cell envelope through electrostatic and other physicochemical interactions. Such interactions can disturb the structural integrity of the cell membrane and increase its permeability, thereby promoting leakage of essential intracellular constituents.

In addition, partial release of Zn²⁺ and Mn²⁺ ions from the Zn/Mn co-doped MOF under biological conditions may contribute to the observed antibacterial activity. These metal ions may interact with membrane-associated components and intracellular biomolecules, resulting in disruption of essential cellular functions. The presence of Zn and Mn centres may also contribute to oxidative stress through the possible generation or accumulation of ROS. Excessive ROS can induce oxidative damage to membrane lipids, proteins, and nucleic acids, thereby impairing cellular integrity and metabolic activity.

The combined effects of membrane disruption, metal-ion-mediated interactions, and oxidative stress may subsequently interfere with vital bacterial processes, including enzyme activity, protein synthesis, cellular respiration, and DNA integrity. These cumulative effects can lead to leakage of intracellular components, loss of cellular homeostasis, inhibition of bacterial growth, and ultimately cell death [74,75].

The antibacterial response may also differ between *Staphylococcus aureus* and *Escherichia coli* because of differences in their cell-envelope architecture. The thick peptidoglycan layer of Gram-positive *S. aureus* and the additional outer membrane containing lipopolysaccharides in Gram-negative *E. coli* may influence the extent of interaction between the MOF particles and bacterial cells. Therefore, the Zn/Mn co-doped MOF is proposed to exert its antibacterial effect through a synergistic combination of direct MOF–cell interaction, possible metal-ion release, membrane destabilization, and oxidative damage.

#### 4.2.3 Antioxidant activity

The DPPH free radical scavenging assay depicted in the Figure S7 indicated that all synthesized MOFs exhibited measurable antioxidant activity, although the extent of scavenging varied among the samples and with increasing concentration. The comparatively higher scavenging observed for the co-doped sample may be associated with the combined influence of Zn and Mn incorporation, which could alter the surface chemistry and availability of redox-active sites within the ZIF-67 framework[76].

### 4.3 Cytotoxicity study

The MTT assay is a widely used colorimetric technique that measures cell metabolic activity by measuring the reduction of the yellow tetrazolium salt (MTT) to purple formazan crystals catalyzed by mitochondrial dehydrogenases in viable cells. This assay provides a reliable quantitative estimation of cell viability and is a standard method for assessing the cytotoxic effects of chemical compounds and nanomaterials. Cells were treated with increasing concentrations of the samples (50, 100, and 250 µg/mL), and cytotoxicity was measured as the percentage of viable cells relative to untreated controls. The half-maximal inhibitory concentration (IC_50_), representing the concentration at which 50% of the cells are inhibited, was calculated for each sample to provide a comparative measure of cytotoxic potency.

The results suggest that both doping and co-doping strategies can modulate the cytotoxic behaviour of MOF materials, potentially allowing for tunable biological activity. The dose-dependent increase in cytotoxicity aligns with typical MTT assay trends, reflecting the progressive impairment of mitochondrial function and cell viability at higher concentrations. The results obtained are tabulated in Table S8.

#### 4.3.1 AO-EB staining

While EB only penetrates cells with damaged membranes, staining their nuclei orange-red and acting as a dependable marker of late apoptosis or necrosis, AO selectively penetrates both live and early apoptotic cells in this assay, intercalating into nuclear DNA and emitting bright green fluorescence. In line with a healthy population of live cells with intact membranes and low basal cytotoxicity, the untreated control showed consistently strong green fluorescence.

Treatment with the undoped Z sample, on the other hand, produced a little but discernible departure from the control. It caused minor plasma membrane instability in a subset of cells, as evidenced by the appearance of scattered red-fluorescent nuclei, even though the majority of cells remained green, indicating comparatively intact viability. This implies that the undoped material has a limited but intrinsic cytotoxic potential, probably not enough to cause widespread apoptotic signalling on its own. After treatment with the doped samples, ZZ and ZM, a more noticeable change in fluorescence patterns was seen. Both substances caused a significant drop in green-stained cells and an increase in red fluorescence intensity. Significant membrane breakdown and higher percentages of late apoptotic or necrotic cells are reflected in the elevated EB absorption. This suggests that, in comparison to the undoped Z formulation, the inclusion of dopants significantly increases the biological effect, whether through structural alteration, greater reactivity, or higher creation of intracellular stress. The merged images for these treatments show a lot of yellow and orange areas, which indicate transitional apoptotic states and validate the doped systems increased apoptotic efficacy, as observed in Fig. 5(a).

This dramatic change indicates a synergistic effect between the dopants and the MOF framework, leading to a markedly increased ability to induce irreversible cytotoxicity, initiate apoptotic pathways, and disrupt cellular homeostasis.

#### 4.3.2 Hoechst staining

Hoechst staining was performed to investigate nuclear morphology and chromatin integrity in cells treated with the undoped (Z) and doped (ZZ, ZM, and ZMZ) ZIF-based samples. Hoechst dyes selectively bind to the minor groove of DNA, predominantly at A–T-rich regions, and emit bright blue fluorescence when excited with ultraviolet light. This staining technique enables sensitive detection of nuclear condensation, chromatin fragmentation, and apoptotic bodies hallmarks of early and late stages of programmed cell death. Thus, analysis of the fluorescence patterns provides valuable insight into the degree of nuclear damage induced by each formulation. The untreated control cells exhibited round, uniformly stained nuclei with smooth edges and diffuse, evenly distributed blue fluorescence. This morphology is characteristic of healthy, viable cells with intact chromatin architecture and minimal nuclear stress. No evidence of condensation or fragmentation was observed, confirming normal cell homeostasis [77].

Numerous cells showed bright, shrunken, and irregularly shaped nuclei, along with distinct nuclear fragmentation, features indicative of advanced apoptosis. The presence of condensed apoptotic bodies indicates that doping enhances the material’s ability to induce nuclear damage. Both ZZ and ZM treatments produced a markedly higher apoptotic index than the undoped Z sample, suggesting that dopant incorporation strengthens the interaction between the materials and cellular components, thereby amplifying nuclear destabilisation.

The nuclear damage in ZMZ-treated cells was significantly more severe and widespread than in any other treatment group, indicating that co-doping produces a synergistic effect that dramatically enhances the pro-apoptotic behaviour of the dual doped sample. This suggests that the physicochemical modifications introduced by co-doping substantially increase the material's ability to trigger chromatin disintegration and irreversible cell death. Collectively, hoechst staining across all treatment groups reveals a clear, progressive pattern of nuclear damage in the order: Control < Z (undoped) < ZZ ≈ ZM < ZMZ (co-doped).

The results strongly support the conclusion that doping particularly co-doping significantly enhances the cytotoxic and apoptosis-inducing properties of ZIF-based materials. ZMZ stands out as the most potent formulation, demonstrating extensive chromatin condensation and nuclear fragmentation. These findings provide compelling evidence for the superior anticancer potential of the co-doped sample and justify its therapeutic potential [78]

### 4.4 Cytotoxicity of the MOFs on BEAS-2B lung epithelial cells

The cytotoxicity evaluation on normal BEAS-2B bronchial epithelial cells showed a concentration-dependent decrease in cell viability for all the tested MOFs. However, the inhibitory concentrations required to achieve comparable cytotoxic effects were higher than those previously observed in lung cancer cells, indicating lower susceptibility of normal cells to treatment. This differential response indicates selectivity toward cancer cells while minimizing toxicity to normal lung epithelial cells. Among the tested formulations, the Z formulation exhibited the highest cytotoxic potential in BEAS-2B cells.

Cytotoxicity of the MOFs on BEAS-2B lung epithelial cells is indicated in Table S8. Values are represented as Mean ± SD (*n* = 3). ns: Non-significant, *p < 0.05, **p < 0.01 compared to Z.

### 4.5 Evaluation of apoptotic protein expression by western blotting

The expression pattern of the studied protein markers is indicated in Fig. 5(b). The altered expression profile of apoptosis-related proteins suggests that the synthesized MOFs modulate apoptotic signalling in treated cells. The pronounced increase in BAX together with the reduction in Bcl-2 indicates a shift toward a pro-apoptotic state, thereby favouring mitochondrial membrane permeabilization and initiation of programmed cell death. The superior response observed for the Zn, Mn-codoped ZIF-67 sample may be attributed to the synergistic effects of dual-metal incorporation, which could enhance intracellular oxidative stress and activate apoptosis-related pathways more effectively than the single-doped materials. Overall, these findings demonstrate that the developed MOFs possess the ability to regulate key apoptotic proteins and contribute to apoptosis induction in cancer cells[79].

### 4.6 Mechanism of cytotoxicity

The possible mode of action of the Zn/Mn co-doped ZIF-67 nanoparticles against lung adenocarcinoma cells is illustrated in Figure S9. Owing to their nanoscale dimensions, these particles may interact efficiently with the cell surface and subsequently be taken up through endocytic processes. Following internalization, the mildly acidic intracellular environment could gradually influence the stability of the ZIF-67 framework, facilitating the release of small amounts of Zn²⁺ and Mn²⁺ ions.

The released metal ions, together with direct interactions between the nanoparticles and intracellular components, may interfere with normal cellular functions. Such disturbances could affect membrane integrity, mitochondrial activity, and other essential biochemical processes, eventually reducing cell viability. Although the precise molecular pathway was not investigated in the present study, the observed cytotoxic response is consistent with the possibility that multiple intracellular events contribute to the loss of cellular function and subsequent cell death. Further mechanistic investigations will be necessary to identify the dominant pathways responsible for the anticancer activity of the Zn/Mn co-doped ZIF-67 nanoparticles.

## 5 Conclusion

The BET isotherms reveal apparent variations in surface area and pore accessibility upon metal doping: Mn incorporation reduces the inherent porosity of ZIF-67, whereas Zn doping induces significant pore blocking and partial framework densification, leading to more reduction in surface area. XRD patterns also support these observations, showing well-defined crystalline reflections for pristine and Mn-doped samples. In contrast, Zn-doped counterparts display peak broadening and intensity loss, indicative of slight structural. FTIR spectra gives evidence for the retention of characteristic imidazolate vibrations across all samples, with slight shifts reflecting successful metal–ligand coordination during doping. TGA profiles demonstrate multistep thermal decomposition typical of ZIF structures, with Mn-doped samples exhibiting enhanced thermal stability and Zn-doped samples showing reduced stability due to defect formation.

SEM and DLS investigations collectively verify the controlled nanoscale architecture and dispersion behaviour of the engineered MOF systems. Electron microscopy revealed well-defined particle morphologies with progressive surface texturing induced by modification, while maintaining structural integrity. Consistently, DLS measurements reveal good hydrodynamic sizes with narrow to moderate polydispersity, indicating colloidal behaviour in aqueous environments depicted by the zeta potential values. The close correspondence between solid-state morphology and solution-phase size highlights the effectiveness of surface engineering in regulating particle characteristics. The doped samples showed excellent antibacterial activity and also showed improved radical scavenging activity with increase in concentration, which is notable, as current literature does not report a standardized quantitative antioxidant capacity for pristine ZIF-67 using any conventional assays.

The cytotoxic efficacy of the newly developed materials was preliminarily evaluated using the A549 human lung adenocarcinoma cell line to assess the induction of apoptotic or necrotic cell death. Cell viability was quantified through the MTT assay, which is based on the metabolic activity of viable cells. In this assay, intracellular NAD(P)H-dependent oxidoreductase enzymes reduce the yellow tetrazolium salt MTT [3-(4,5-dimethylthiazol-2-yl)-2,5-diphenyltetrazolium bromide] into insoluble formazan crystals. The resulting formazan product is subsequently solubilized in dimethyl sulfoxide (DMSO), producing a purple-coloured solution that exhibits a characteristic absorbance at 570 nm. The measured absorbance is directly correlated with the number of metabolically active cells, thereby serving as an indicator of cell viability.

In conclusion, the MTT assay demonstrated that co-doped ZMZ exhibits superior cytotoxicity compared to the undoped and singly doped samples, highlighting the importance of compositional tuning in designing biologically active MOF materials. These findings provide valuable insights for the rational design of MOFs for cytotoxic and therapeutic applications. Collectively, the fluorescence staining results reveal a clear trend in which cytotoxicity increases progressively from the undoped Z sample to the doped samples, with ZMZ emerging as the most biologically active formulation. These findings highlight the crucial role of doping in modulating the physicochemical and biological properties of MOF-based systems. The conclusion that structural alteration of the MOF enhances its interaction with cellular components and increases apoptotic induction is supported by the observed increase in red fluorescence in the doped samples. Overall, the AO/EB assay provides strong evidence that the doped MOF samples, especially ZMZ, have strong anticancer potential, making them attractive options for mechanistic and therapeutic research.

## CRediT authorship contribution statement

**Preema Cealla Pais:** Writing – review & editing, Writing – original draft, Methodology, Investigation, Formal analysis, Data curation, Conceptualization. **Ronald Aquin Nazareth:** Writing – review & editing, Supervision, Resources, Project administration, Funding acquisition. **Ashwini Prabhu:** Writing – review & editing, Writing – original draft, Validation, Investigation, Formal analysis, Data curation, Conceptualization. **Sudhakar Y N:** Writing – review & editing, Resources, Project administration. **Arnab Parui:** Writing – review & editing, Writing – original draft, Methodology, Investigation.

## Declaration of competing interest

The authors declare the following financial interests/personal relationships which may be considered as potential competing interests:

Ronald Aquin Nazareth reports a relationship with Mangalore Jesuit Educational Society (MJES) that includes: funding grants. Ronald Aquin Nazareth has patent pending to St. Aloysius (Deemed to be University). If there are other authors, they declare that they have no known competing financial interests or personal relationships that could have appeared to influence the work reported in this paper.

## Data Availability

Data will be made available on request.
