# Supplementary material for: ZIF-67 and Mn & Zn co-doped ZIF-67 as multifunctional therapeutic platforms: Antibacterial, antioxidant, and anticancer assessment in lung cancer cells in vitro
Source: Biomater Biosyst. 2026 Sep 19;24:100148. doi: 10.1016/j.bbiosy.2026.100148 (PMC13633908; doi:10.1016/j.bbiosy.2026.100148)
Supplement: Supplementary file 1 [file mmc1.docx]

**ZIF-67 and Mn & Zn co-doped ZIF-67 as Multifunctional Therapeutic Platforms: Antibacterial, Antioxidant, and Anticancer Assessment in Lung Cancer Cells In Vitro**

Preema Cealla Pais^1^, Ronald Aquin Nazareth ^1^*, Ashwini Prabhu^2,3^, Sudhakar Y N^4^#, Arnab Parui^2^

^1^Department of Chemistry, St. Aloysius (Deemed to be University), Mangaluru 575003.Karnataka, India

^2^Division of Cancer Research and Therapeutics (CaRT), Yenepoya Research Centre, Yenepoya (Deemed to be University), Mangalore 575 018, India

^3^Central Research Laboratory, Srinivas Institute of Medical Sciences and Research Centre, Srinivas University, Mangalore 574146, India

^4^Manipal Institute of Technology, Manipal Academy of Higher Education, Manipal, India

Corresponding author's email: *ronald.nazareth@staloysius.edu.in; #sudhakar.yn@manipal.edu

***BET Studies***

Figure S1. BET graphs of (a) ZIF 67, (b)Zn-doped ZIF 67, (c)Mn-doped ZIF 67, (d)Zn/Mn co-doped ZIF 67 respectively. [inset (BJH plots)]

**Table S1: The BET and BJH parameters for ZIF-67 and its doped samples**

| **Sample Code** | **BET surface area**  **(m^2^g^-1^)** | **BET pore size (nm)** | **BET pore volume (cm^3^g^-1^)** | **BJH surface area (m^2^g^-1^)** | **BJH pore size (nm)** | **BJH pore volume (cm^3^g^-1^)** |
| --- | --- | --- | --- | --- | --- | --- |
| Z | 1227.5 | 2.10 | 0.645 | 704.9 | 1.40 | 0.2481 |
| ZZ | 130.9 | 2.08 | 0.068 | 0.957 | 1.32 | 0.0006 |
| ZM | 283.02 | 2.45 | 0.173 | 243.29 | 1.58 | 0.096 |
| ZMZ | 4.22 | 10.71 | 0.011 | 10.33 | 4.81 | 0.012 |

***Average particle size analysis***

**Figure S2.** Average particle size (APS) analysis using TEM of the synthesised samples (a) ZIF 67, (b) Zn-doped ZIF 67, (c) Mn-doped ZIF 67, (d) Zn/Mn co-doped ZIF 67s respectively

***Dynamic light scattering (DLS) analysis:***

**Figure S3.** Dynamic Light Scattering (DLS) analysis of (a) ZIF 67, (b) Zn-doped ZIF 67, (c) Mn-doped ZIF 67, (d) Zn/Mn co-doped ZIF 67 showing the hydrodynamic size distribution of the synthesised nanoparticles measured in aqueous medium at 25 °C.

Table S2: The experimental zeta potential values of ZIF-67 and its single and dual doped samples

| **Samples** | **Zeta Potential** |
| --- | --- |
| **Z** | **-13.43 ±1.47 mV** |
| **ZZ** | **-44.7 ± 2.47 mV** |
| **ZM** | **-34.43 ± 1.58 mV** |
| **ZMZ** | **-11.20 ± 1.59 mV** |

Table S3: The Elemental compositions of ZIF-67 and its single and dual doped samples

| **Element** | **Z** | **ZZ** | **ZM** | **ZMZ** |
| --- | --- | --- | --- | --- |
| C (% wt.) | 59.15 | 35.02 | 55.42 | 47.41 |
| O (% wt.) | - |  | 10.25 | 15.72 |
| N (% wt.) | 15.62 | 21.71 | 18.75 | 17.74 |
| Co (% wt.) | 25.22 | 18.61 | 15.16 | 13.58 |
| Zn (% wt.) | - | 9.56 | - | 5.19 |
| Mn (% wt.) | - | - | 0.42 | 0.36 |

***XPS Studies***

Figure S4.XPS spectra of the dual doped material ZMZ (a) Survey spectrum, (b) Spectrum of C 1s (c) Spectrum of O 1s (d) Spectrum of N 1s (e) Spectrum of Co 2p, (f) Spectrum of Mn 2p, and (g) Spectrum of Zn 2p

Table S4: ICP OES data of ZIF-67 and its doped samples

| Sample | Co (ppm) | Zn (ppm) | Mn (ppm) | Co (mol%) | Zn (mol%) | Mn (mol%) | Metal ratio |
| --- | --- | --- | --- | --- | --- | --- | --- |
| Pristine ZIF-67 | 4.655±0.06 | - | - | 100 | - | - | Co_1.00_ |
| Zn/Mn co-doped ZIF-67 | 4.441±0.05 | 0.161±0.003 | 0.473±0.007 | 85.39 | 2.85 | 9.96 | Co_1.00_ Zn_0.033_ Mn_0.138_ |

*Table S5: Zone of inhibition (mm) against the test organisms*

| **Name of the Bacteria** | **Z** | **ZZ** | **ZM** | **ZMZ** |
| --- | --- | --- | --- | --- |
| *Staphylococcus aureus* | 0±0.0 mm | 8±0.2 mm | 14±0.4 mm | 16±0.3 mm |
| *Escherichia coli* | 9±0.2 mm | 9±0.3 mm | 11±0.2 mm | 15±0.1 mm |

(b)

**(a)**

Figure S5. Antimicrobial activity of samples against (a) *Staphylococcus aureus* and (b) *Escherichia coli*. Samples: (Z) ZIF 67, (ZZ) Zn-doped ZIF 67, (ZM) Mn-doped ZIF 67, (ZMZ) Zn/Mn co-doped ZIF 67, PC: Positive Control VC: Vehicle Control DMF

Figure S6. Antimicrobial activity by 96 well microdilution method

Table S6: Minimum Inhibitory Concentration and Minimum Bactericidal Concentration of the samples against *Staphylococcus aureus* and *Escherichia coli*

| Sample | *S. aureus* MIC (mg/mL) | *S. aureus* MBC (mg/mL) | *E. coli* MIC (mg/mL) | *E. coli* MBC (mg/mL) |
| --- | --- | --- | --- | --- |
| Z | 0.5 | >1.0 | 0.03125 | 0.25 |
| ZZ | 0.5 | >1.0 | 0.0625 | 1.0 |
| ZM | 0.25 | >1.0 | 0.0625 | 1.0 |
| ZMZ | 0.25 | >1.0 | 0.25 | >1.0 |

Table S7: (a) Antibacterial effect of different MOF-based compounds against *E. coli*.

(*–this work)

| Material | MIC (μg/mL) | MBC (μg/mL) | Ref. |
| --- | --- | --- | --- |
| Ag NWs@H-ZIF-67 | 12 dark; 9 under light | - | [72] |
| Ag NWs@C-HZ67 | 12 dark; 9 under light | - | [72] |
| ZIF-67 | 31.25 | 250 | * |
| Zn doped ZIF-67 | 62.5 | 1000 | * |
| Mn doped ZIF-67 | 62.5 | 1000 | * |
| Zn/Mn codoped ZIF-67 | 250 | >1000 | * |

Table S7: (b) Antibacterial effect of different MOF-based compounds against *S. aureus* (*–this work)

| Material | MIC (μg/mL) | MBC (μg/mL) | Ref. |
| --- | --- | --- | --- |
| Ag@ZIF-67 | 3.9 | - | [73] |
| Ag NWs@H-ZIF-67 | **30 (**dark);  **12 (**under light) | - | [72] |
| Ag NWs@C-HZ67 | 15 (dark); 3 (under light) | - | [72] |
| ZIF-67 hollow spheres | 6.25 | - | [74] |
| ZIF-67 | 500 | >1000 | * |
| Zn doped ZIF-67 | 500 | >1000 | * |
| Mn doped ZIF-67 | 250 | >1000 | * |
| Zn/Mn codoped ZIF-67 | 250 | >1000 | * |

***Antioxidant activity***

Figure S7. DPPH free radical scavenging activity of the pristine and doped MOF samples (n=3)

Table S8**:** Cytotoxicity of the MOFs on A549 lung cancer cells. Values are represented as Mean ± SD (n=3). ns: Non-significant, **p*<0.05, ***p*<0.01 compared to Z

| **Sample Code** | **Concentration(μg/ml)** | **Cytotoxicity (%)** | **IC50** |
| --- | --- | --- | --- |
| Z | 50 | 17.80 ± 8.08 | 130.24 |
|  | 100 | 48.58 ± 2.09 |  |
|  | 250 | 86.60 ± 1.47 |  |
| ZZ | 50 | 7.88 ± 4.70 | 132.97^ns^ |
|  | 100 | 54.24 ± 7.95 |  |
|  | 250 | 88.26 ± 2.08 |  |
| ZM | 50 | 2.17 ± 0.79 | 123.11*** |
|  | 100 | 51.29 ± 10.39 |  |
|  | 250 | 86.11 ± 1.43 |  |
| ZMZ | 50 | 24.56 ± 1.12 | 94.89*** |
|  | 100 | 67.87 ± 2.45 |  |
|  | 250 | 91.34 ± 4.32 |  |

***Hoechst Staining***

Figure S8. Hoechst staining images of control, (a) ZIF 67, (b) Zn-doped ZIF 67, (c)Mn-doped ZIF 67, (d) Zn/Mn co-doped ZIF 67, respectively.

Figure S9. A schematic representation of Zn/Mn Codoped ZIF- 67 MOF-induced cytotoxicity in A549 lung adenocarcinoma cells.
